# The Rab11 Effector Protein FIP1 Regulates Adiponectin Trafficking and Secretion

**DOI:** 10.1371/journal.pone.0074687

**Published:** 2013-09-11

**Authors:** Brian P. Carson, Josep Maria Del Bas, Jose Maria Moreno-Navarrete, Jose Manuel Fernandez-Real, Silvia Mora

**Affiliations:** 1 Department of Cellular and Molecular Physiology, Institute of Translational Medicine, the University of Liverpool, Liverpool, United Kingdom; 2 Section of Diabetes, Endocrinology and Nutrition, Hospital Dr. Josep Trueta, Girona, Spain; Graduate School of Medicine, the University of Tokyo, Japan

## Abstract

Adiponectin is an adipokine secreted by white adipocytes involved in regulating insulin sensitivity in peripheral tissues. Secretion of adiponectin in adipocytes relies on the endosomal system, however, the intracellular machinery involved in mediating adiponectin release is unknown. We have previously reported that intracellular adiponectin partially compartmentalizes with rab 5 and rab11, markers for the early/sorting and recycling compartments respectively. Here we have examined the role of several rab11 downstream effector proteins (rab11 FIPs) in regulating adiponectin trafficking and secretion. Overexpression of wild type rab11 FIP1, FIP3 and FIP5 decreased the amount of secreted adiponectin expressed in HEK293 cells, whereas overexpression of rab11 FIP2 or FIP4 had no effect. Furthermore shRNA-mediated depletion of FIP1 enhanced adiponectin release whereas knock down of FIP5 decreased adiponectin secretion. Knock down of FIP3 had no effect. In 3T3L1 adipocytes, endogenous FIP1 co-distributed intracellularly with endogenous adiponectin and FIP1 depletion enhanced adiponectin release without altering insulin-mediated trafficking of the glucose transporter Glut4. While adiponectin receptors internalized with transferrin receptors, there were no differences in transferrin receptor recycling between wild type and FIP1 depleted adipocytes. Consistent with its inhibitory role, FIP1 expression was decreased during adipocyte differentiation, by treatment with thiazolidinediones, and with increased BMI in humans. In contrast, FIP1 expression increased upon exposure of adipocytes to TNFα. In all, our findings identify FIP1 as a novel protein involved in the regulation of adiponectin trafficking and release.

## Introduction

Adipose tissue has now been recognized as an endocrine organ, producing and releasing a number of hormones termed adipokines that regulate metabolism and energy homeostasis. Adiponectin is one such adipokine secreted exclusively by adipocytes [1–3], that functions *in vivo* as an insulin sensitizer [4–6], reducing glucose production by the liver [7] and enhancing fatty acid oxidation in skeletal muscle [6]. Adiponectin synthesis and secretion is compromised in obesity and diabetes, resulting in decreased circulating serum levels [8,9].

In eukaryotic cells, intracellular vesicle traffic and secretion are complex multi-step processes that are regulated by a diverse number of proteins in different pathways. Secretory cargo destined for exocytosis may traffic either directly from the trans-Golgi network to the plasma membrane (the ‘constitutive’ pathway), can be packaged to a storage compartment (‘regulatory compartment’) or can involve the endosomal compartment of the cell [10–13]. Studies in a range of cell types and with a variety of secretory cargo have suggested that the route to the cell surface can vary, depending on both the cargo and the cell type. Despite the important role that adiponectin plays in regulating metabolism, the mechanisms that regulate its production, trafficking and secretion are still poorly understood.

Adiponectin is initially synthesized in the lumen of the endoplasmic reticulum (ER) following the removal of a short signal peptide. The protein moves through the Golgi and *trans*-Golgi network where an important pool of adiponectin is packaged into GGA1 (Golgi localizing γ-adaptin ear homology domain ADP ribosylating factor -ARF- binding) coated vesicles [14] and delivered to endosomes [14]. Other adipokines such as adipsin also rely on the endosomal compartment for their release (Clarke et al 2006; Millar et al. 2000), whereas leptin does not (Xie et al 2008). Adiponectin was found to colocalize with rab11, a marker of the Endosomal Recycling Compartment (ERC) [15,16]. The rab11 subfamily of rab GTPases comprises rab11a, rab11b and rab25, which localize to the ERC and regulate endosomal trafficking through this compartment. Currently several rab11-effector proteins have been identified [17]. Among them, the rab11-Family of Interacting Proteins (FIPs) comprise five members: FIP1, FIP2, FIP3 (or Arfophilin), FIP4 (or Arfophilin-2) and FIP5 (also known as Rip11, Gaf-1/Gaf1-b) [18,19]. Despite sharing limited homology, all FIP proteins contain a conserved 20 amino acid rab binding domain (RBD) which allows interaction with rab11. FIPs have been involved in regulating multiple distinct membrane trafficking events in several cell types. For example, FIP2 has a role in regulating the traffic of Aquaporin 2 (AQP2) and the chemokine receptor CXCRS2 [20,21]. In adipocytes, FIP5 has been involved in the translocation of the facilitative glucose transporter Glut4 containing vesicles to the cell surface in response to insulin [22].

In order to better understand the mechanisms that regulate adiponectin trafficking and secretion, we have examined the role of FIPs in this process. Here we have identified rab11 FIP1 as a regulator of adiponectin secretion. We report that overexpression of FIP1 reduces adiponectin secretion whereas FIP1 knock down increases adiponectin release. We also show that FIP1 expression is downregulated during adipogenesis and by treatment with thiazolidinediones, and is inversely correlated with BMI in humans. These findings identify FIP1 as a new player involved in the intracellular trafficking of adiponectin.

## Materials and Methods

### 2.1: Materials and plasmids

HEK293 and 3T3L1 cells were obtained from ATCC.

Tissue culture reagents (FBS, trypsin, penicillin), insulin, IBMX, dexamethasone, puromycin were from Sigma. Adiponectin ELISA kits were from R&D (Minneapolis, Minnesota). The following constructs were used in this study: a vector expressing adiponectin-myc (pcDNA3.1-adiponectin-myc) [14], FIP1GFP (pEGF-C3-FIP1) [24], FIP2-GFP (pEGFP-C1-FIP2) [25], FIP3-GFP (pEGFP-C1-FIP3) [26], FIP4-GFP (pEGFP-C1-FIP4 _82-637_) [27] and FIP5 GFP (pEGFP-C1-Rip11) [28]. All constructs coding for Rab11 FIP proteins were a generous gift from Dr. M. McCaffrey, University College Cork (Ireland). The PKLO.1 puro empty plasmid (cat number SHC001), the PKLO.1 plasmid carrying a non-targeting shRNA (NT shRNA) sequence (cat SHC002) and PKLO.1 puro vectors carrying shRNAs for FIP proteins were all from Sigma (MISSION shRNA). The PMDG2 and PMCV-dR8.74 vectors to generate the lentiviral vectors were a gift from Dr. Antonio Zorzano (IRB, Barcelona). Biotinylated human adiponectin was purchased from R&D; Alexa-488 conjugated transferrin was obtained from Life Technologies.

### 2.2: Human subjects

We studied 233 adipose tissue samples (123 visceral and 110 subcutaneous) from a group of Caucasian subjects with a body mass index (BMI) between 20 and 58 kg/m^2^ recruited at the Endocrinology Service of the Hospital Universitari Dr. Josep Trueta (Girona, Spain). All subjects declared that their body mass had been stable for at least 3 months before the study and gave written informed consent after the purpose, nature and potential risks for the study were explained to them. The experimental protocol was approved by the Ethics Committee of the *Hospital Universitari de Girona Dr. Josep Trueta* (Girona, Spain), so we certify that all applicable institutional regulations concerning the ethical use of information and samples from human volunteers were followed during this research. Adipose tissue samples were obtained from subcutaneous and visceral depots during elective surgical procedures (cholecystectomy, surgery of abdominal hernia, and gastric by-pass surgery), washed, fragmented and immediately flash-frozen in liquid nitrogen before be stored at -80^°^C.

### Anthropometric measurements

BMI was calculated as mass (kg) divided by height squared (m^2^). According to this anthropometric parameter, subjects were classified as: lean (BMI<25 kg/m^2^) or obese (BMI ≥30 kg/m^2^) following World Health Organization guidelines. Serum glucose concentrations were measured in duplicate by the glucose oxidase method using a Beckman glucose analyzer II (Beckman Instruments, Brea, California). Glycosylated haemoglobin (HbA1c) was measured by the high-performance liquid chromatography method (Bio-Rad, Muenchen, Germany, and autoanalyser Jokoh HS-10, respectively). Intraassay and interassay coefficients of variation were less than 4% for all these tests. Total serum triglycerides were measured through the reaction of glycerol-phosphate-oxidase and peroxidase on a Hitachi 917 instrument (Roche, Mannheim, Germany). HDL cholesterol was quantified after precipitation with polyethylene glycol at room temperature.

### 2.2: Cell Culture, treatments and transfections

Cell culture of HEK293 cells and 3T3L1 cells were performed as previously described [14]. Fully confluent 3T3L1 cells were differentiated with insulin, IBMX and dexamethasone as described [14]. 3T3L1 fibroblasts stably expressing shRNA were grown in DMEM media supplemented with 10% calf serum, and 3 µg.ml^-1^ of puromycin. Once confluent, cells were differentiated in regular differentiation media. Transfections: GFP tagged rab11-FIP expression vectors (pEGFP) myc-tagged adiponectin (pcDNA3 vector) or plasmids carrying shRNA sequences for FIP proteins were transfected into HEK293 cells using lipofectamine 2000 (Life Technologies) as per the manufacturer’s instructions. For each FIP protein a pool of shRNA expressing vectors was transfected. The constructs used to deplete FIP1 (rab11 FIP1C also called RCP) targeted all the spliced mRNAs for this protein. Isolated preadipocytes (Zen-Bio Inc., Research Triangle Park, NC, USA) from omental and subcutaneous adipose tissue were cultured and differentiated for 14 days as previously described [29]. *TNF-α* (100 ng/ml) and LPS-stimulated macrophage-conditioned medium (MCM, 5%) treatments were performed during omental and subcutaneous adipocyte differentiation as previously described [29]. Differentiated 3T3L1 adipocytes were treated at day 9 following differentiation with 50 ng/ml TNFα or Interleukin-6 (IL6) or 5% (v/v) macrophage-conditioned media as indicated in the figure legends. Macrophage conditioned media enriched in pro-inflammatory cytokines was obtained after stimulating human THP macrophages or mouse bone marrow-derived macrophages with LPS (10 ng/ml) for 24hr. Hypoxia treatments: Differentiated 3T3L1 adipocytes at day 9 of differentiation were incubated in normoxia (5% CO_2_, 95% air) or under hypoxic conditions at 1% O_2_, 94% N_2_, 5% CO_2_ for 8, 16 or 24hr. For troglitazone (TZD) treatments, differentiated 3T3L1 adipocytes were treated with either the vehicle (DMSO) or the PPARγ agonist (TZD) at a final concentration of 10 µM for 3 days. Adiponectin secretion and gene expression assays are described below.

### 2.3: Lentivirus production and infections

Lentiviral viruses carrying FIP specific shRNAs were generated by cotransfecting HEK293 cells (ATTC collection) with the PKLO.1-puro plasmid (Sigma) and the packaging vectors PMCV-dR8.74 and PMD2G using the calcium phosphate method. Each shRNA construct (MISSION shRNAs from SIGMA) was transfected individually with the packaging vectors to generate lentiviral particles each expressing a single shRNA. The lentiviral particles used as a pool in the study expressed shRNAs that targeted all the splicing forms for FIP1.The media containing lentiviruses were collected 24h and 48h post transfection, centrifuged, the supernatant filtered through a 0.45 µm filter and the media containing virus stored at -80^o^C until use. To generate the stable cell lines, 3T3L1 cells were incubated with polybrene (8 µg.ml^-1^) for 1 hour and infected with all gene specific shRNA containing viruses simultaneously. 24h post-infection the medium containing viruses was removed and replaced with medium containing 3 µg.ml^-1^ of puromycin for the selection of successfully infected cells. Media was changed every 2 days thereafter and cells were maintained in DMEM FBS media, in the presence of 3 µg.ml^-1^ puromycin.

### 2.4: Immunofluorescence

3 T3L1 cells were fixed, permeabilized and immunostained as previously described [14,15]. The following primary antibodies were used: anti-FIP1 (Sigma), Anti-myc (clone 9E10, Santa Cruz Biotechnology), anti-rab11 (Life Technologies), anti-adiponectin (R&D Minneapolis), anti-EEA1 (early endosome antigen1) (a gift from Dr. S. Urbé, University of Liverpool), anti-Glut4 (a gift from Dr. Zorzano, IRB, Barcelona) anti-rab5 (BD Biosciences), Alexa-350, -488 and -594 conjugated antibodies (Life Technologies) and anti-rat Texas Red (Abcam) were used as secondary antibodies. Labelling of transferrin and adiponectin receptors (AdipoR1 and R2): Cells were incubated in biotinylated adiponectin (0.04pg) or transferrin A-594 (5µg.ml^-1^) containing DMEM media for 1 hour at 4^°^C. Media was removed and alexa-488-streptavidin (1:100) was subsequently added for 30 minutes at 4^°^C in the dark. Labelled adiponectin and transferrin receptors were allowed to internalize at 37 ^°^C for 5, 15, 30 or 45 minutes. Cells were then washed twice with PBS on ice and fixed in 4% PFA / PBS for 10 minutes. Cells were mounted and imaged in a Leica DMI6000 microscope equipped with LAF software. Pixel Intensity Spatial Correlation Analysis of colocalisation was carried out using the Fiji plugin for ImageJ Software. Mander’s coefficients of correlation M1 and M2 as well as Pearson’s Coefficient (PC) were used for colocalisation analysis. Green and red channel images of cells were opened by the application and a region of interest incorporating the whole cell was selected using the polygon selection tool. Analysis was performed using the coloc 2 tool with a PSF of 3.0 and 10 Coste’s randomisations. Outputs indicating Mander’s coefficients M1 and M2 and Pearson’s Coefficient (PC) were obtained and reported as Mean ± SEM.

### 2.5: Western blotting

Cells were homogenised in ice-cold lysis buffer (20 mM Tris [pH 7.8], 137 mM NaCl, 2.7 mM KCl, 1 mM MgCl_2_, 1% Triton X-100, 10% [vol/vol] glycerol, 10 mM NaF, 1 mM EDTA, 5 mM sodium pyrophosphate, 0.5 mM Na _3_VO_4_, 1 µg.ml^-1^ leupeptin, 0.2 mM phenylmethyl sulfonyl fluoride, 1 µg.ml^-1^ aprotinin, 1 mM dithiothreitol, 1 mM benzamidine). Samples were incubated at 4^°^C for 20 minutes, centrifuged (14,000 *RPM* for 15 min at 4^°^C) and protein concentration of the supernatant was determined using the Bio-Rad Protein Assay Kit. Samples were separated on a SDS-PAGE, transferred to nitrocellulose membranes, blotted in 5% non-fat milk in Tris-buffered saline (pH 7.6) and subsequently immunoblotted with primary and secondary antibodies. Membranes were washed in TBS -0.1% tween buffer and visualized using chemiluminescence reagent (Geneflow). Anti-FIP antibodies: anti-FIP1 antibody was from Sigma, anti-FIP3 and anti-FIP5 antibodies were a generous gift from Dr. Mary McCaffrey (UCC, Ireland) [23,26]. The anti-β-tubulin antibody was from Sigma.

### 2.6: Glucose uptake

Fully differentiated 3T3L1 adipocytes were serum deprived for 3 hours in Dulbeco Modified Eagles Media (DMEM). Cells were then incubated in glucose depleted uptake media (20 mM Hepes pH=7.0, 137 mM NaCl, 4.7mM KCl, 1.2 mM MgSO_4,_ 1.2 mM KH_2_PO_4_, 2.5 mM CaCl_2_) for one hour. Insulin at 100nM final concentration was added for 30 minutes and uptake was carried out for 10 minutes in uptake solution supplemented with 1 mM 2-deoxyglucose and ^3^H-2-deoxyglucose (American Radiolabeled chemicals, Inc) at 1 µCi.ml^-1^. Following the uptake cells were washed 4 times in ice-cold PBS containing 50 mM cold glucose and lysed in 0.5N NaOH and an aliquot of the lysate (200 µl) quantified for ^3^H-2-D-deoxyglucose and another (10 µl) used for protein quantification.

### 2.7: Secretion studies

Released and intracellular adiponectin content was determined using an ELISA kit (R&D, Minessota) following the manufacturer’s instructions. The portion of secreted adiponectin was expressed as percentage of the total adiponectin (media plus lysate).

### 2.8: Transferrin recycling

Transferrin recycling was assayed using fluorescence microscopy and flow cytometry as previously described [30]. Briefly, for transferrin uptake: 3T3L1 fibroblasts were first incubated for 30 min at 37°C in serum-free DMEM medium. Cells were trypsinized, incubated in DMEM media containing Alexa-488 conjugated transferrin (5 µg.ml^-1^) for 10 min at 4°C, and then shifted to 37°C, and incubated for various times as indicated. The cells were then pelleted at 4°C, washed once in ice cold PBS and once in a buffer containing: 0.1 M glycine, 150 mM NaCl, pH=3.0. Cells were resuspended in PBS containing 1% BSA and fluorescence quantified by flow cytometry. For transferrin release: cells were loaded for 45 min with transferrin at 37°C and at different time points transferred to ice and washed in PBS/acid wash buffer as above. Cells were resuspended in PBS containing 1% BSA and fluorescence quantified by flow cytometry, using a FACS Calibur, (Becton Dickinson, San Jose, CA). Data were acquired and analyzed by using the BD CellQuest^TM^Pro Software (BD Biosciences, Heidelberg, Germany). The geomean was used to plot data.

### 2.9: RNA extraction and Real time quantitative PCR. Cellular studies

Total RNA was isolated using Tri reagent (Sigma) following the manufacturer’s instructions. RNA was quantitated by spectrophotometry and visualized in an agarose gel. Total RNA was reverse transcribed to cDNA using Superscript VILO reverse transcription kit (Life Technologies) as per the maufacturer’s instructions. qPCR Primers were designed using Primer3 Input (v.0.4.0) (http://frodo.wi.mit.edu/primer3/). Primers were then checked for self-complementarity and primer dimer formation by running in an agarose gel. The efficiency of primers was then optimized for concentration and melting temperature on a Step 1 Plus instrument (Applied Biosystems). Samples were run in triplicate and relative quantification was carried out using the ∆∆Ct method with Cyclophilin A as an internal control. The primers used on these studies were: For mouse FIP1: Forward 5’–3’: GCTTCAGGAGGAAGCATTTG; Reverse 5-3’:CGGTAGGATGGCAAAGACAT; For Cyclophilin A : Forward: 5’–3’: GCATACAGGTCCTGGCATCT; Reverse 5’–3’: TTACAGGACATTGCGAGCAG. For Glut4: forward: 5’–3’: TTCACGTTGGTCTCGGTGCTCTTA, Reverse 5’–3’: CCACAAAGCCAAATATGGCCACGA; For Glut1: Forward 5’–3’: TCAACGAGCATCTTCGAGAAGGCA; Reverse 5’–3’: TCGTCCAGCTCGCTCTACAACAAA.

For the human studies: RNA was isolated using the RNeasy Lipid Tissue Mini Kit (QIAgen). The integrity of each RNA sample was checked by Agilent Bioanalyzer (Agilent Technologies, Palo Alto, CA). Total RNA was reverse transcribed to cDNA using High Capacity cDNA Archive Kit (Applied Biosystems) according to the manufacturer’s protocol. Real-time quantitative PCR was conducted using Light Cycler 480 Probes Master (Roche) using pre-validated TaqMan primer/probes as follows: endogenous control PPIA (4333763, cyclophilin A) and target genes such as RAB11 family interacting protein 1 (RAB11FIP1, Hs00951195_m1), peroxisome proliferator-activated receptor gamma (PPARγ, Hs00234592_m1), adiponectin (ADIPOQ, Hs00605917_m1), leptin (LEP, Hs00174877_m1), Insulin receptor substrate 1 (IRS-1, Hs00178563_m1) and fatty acid synthase (FASN, Hs00188012_m1). Relative quantification was carried out using the ∆∆Ct method using PPIA gene expression as an internal control.

### 2.10: Statistical analysis

Statistical analyses were carried out using GraphPad Prism4 software (GraphPad software) and One way ANOVA analyses were carried out with a confidence interval of 95% and statistical significance was considered if *p* < 0.05. For the human studies a T test was performed using the SPSS 12.0 software for Windows (SPSS, Chicago, IL, USA) was used. All assays were performed at least in duplicate and reported as mean ± SEM. The comparison between groups was performed using two-way ANOVA followed by post-hoc analysis (using Bonferroni post hoc tests). The relation between variables was analyzed by bivariate correlation (Spearman’s test). For Pixel Intensity Spatial Correlation Analysis of colocalization, SPSS 19.0 software for Windows (SPSS, Chicago, IL, USA) was used to carry out independent T-tests and One Way ANOVA where appropriate with statistical significance considered if *p* < 0.05.

## Results

### 3.1: Expression/ knockdown of rab11 FIP1 alter adiponectin secretion in HEK 293 cells

**Figure 1:**
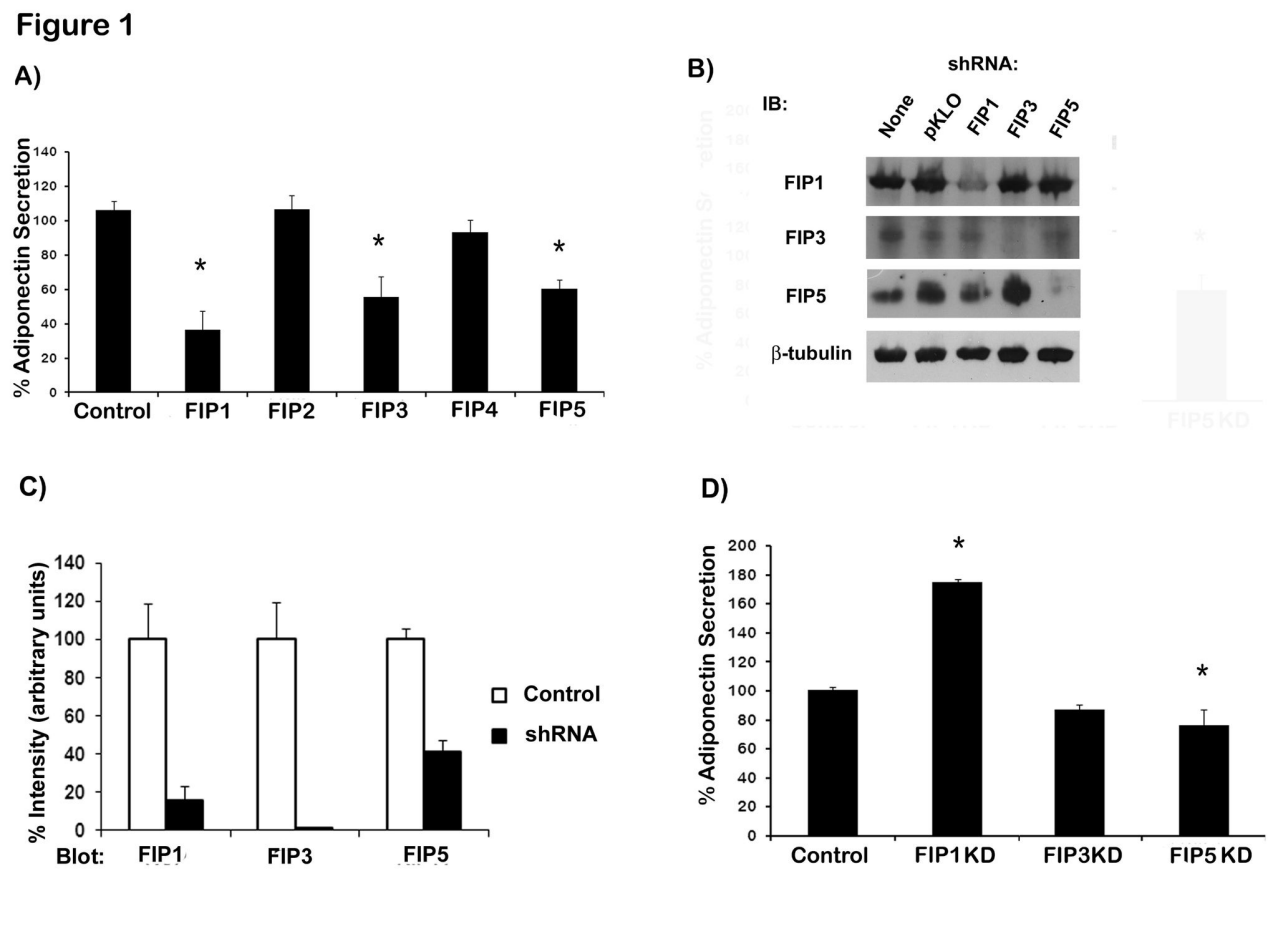
Rab11 FIP proteins regulate adiponectin release in HEK293 cells. **A**) GFP tagged wild type RAB11-FIPs and myc tagged adiponectin were transiently co-transfected in HEK293 cells. 24hr following transfection adiponectin was quantitated by ELISA as indicated in the materials and methods section **B**) Knock down of FIP proteins in HEK293 cells. HEK293 were left untransfected or transfected with a shRNA empty vector (pKLO.1puro) or transfected with a pool of plasmid expressing shRNA sequences specific for FIP proteins as indicated. Whole cell lysates were obtained and protein samples were separated on SDS-PAGE, transferred to nitrocellulose filters and immunoblotted with specific antibodies detecting the human isoforms of FIP1, FIP3 and FIP5 proteins. Anti β-tubulin antibody was used as loading control. **C**) Quantification of the FIP knockdown efficiency for each FIP protein. Data are mean ± SEM from three independent experiments. **D**) HEK293 were transfected with a shRNA expression (pKLO.1puro) empty vector or shRNA vectors containing shRNA sequences for FIP proteins (as indicated, mission shRNA SIGMA). Cells were selected for 96 hours in puromycin (2 µg/ml) containing media and surviving cells were transfected with a plasmid coding for adiponectin-myc. 24hr following transfection adiponectin was quantitated by ELISA in cell lysates and media and adiponectin secretion was calculated as indicated in the materials and methods section. Data are means ± SEM of at least three independent experiments Statistical analysis: One way ANOVA * indicates statistically different from control cells (p < 0.05).

We have previously demonstrated that intracellular adiponectin partially colocalizes with rab11 in adipocytes and expression of rab11 mutants inhibited the secretion of adiponectin [15]. To further characterize the intracellular compartmentalization and trafficking of intracellular adiponectin, we investigated the role of rab11 FIP effectors in adiponectin release in HEK293 cells. We chose to carry out the initial screening in HEK293 cells because of our previous experience with these cells in adiponectin secretion studies and the fact that they are easy to transfect. To this end, we first co-expressed plasmids coding for adiponectin-myc and wild type GFP-tagged FIPs into HEK293 cells. Transfection efficiency in these cells was approximately 70% as determined by GFP expression assessed by fluorescent microscopy (data not shown). Similar levels of GFP-FIP proteins were achieved from the expression of FIP-GFP plasmids as determined by immunoblotting (Figure S1). As shown in Figure 1A, expression of wild type FIP1, FIP3 and FIP5 significantly reduced adiponectin release in HEK293 cells whereas expression of FIP2 or FIP4 had no effect on adiponectin secretion.

To further confirm the involvement of these rab11-FIP proteins in adiponectin secretion we knocked down the expression of FIP1, FIP3 or FIP5 in HEK293 cells by expressing a pool of plasmids that carried shRNA specific sequences for each FIP gene. Following transfection, cells were selected in the presence of puromycin to eliminate cells that did not incorporate the shRNAs and selected cells were subsequently transfected with a plasmid coding for adiponectin-myc. Cells were allowed to recover for 24 hrs and adiponectin secretion was measured by ELISA. The efficacy and specificity of each FIP knockdown was assessed by immunoblotting of cellular lysates with specific antibodies against each FIP isoform (Figure 1B). Effective downregulation was achieved for all FIP proteins. Data from quantification of several observations indicated a reduction at the protein level of 60% for FIP5, 80% for FIP1 and 95% for FIP3 (Figure 1C). We found that FIP1 (Rab11 FIP1C) depletion strongly enhanced adiponectin secretion, whereas knock down of FIP5 only mildly decreased adiponectin release and FIP3 knock down had no effect on adiponectin secretion (Figure 1D). To confirm that the effects on adiponectin secretion are a bona fide consequence of the knock down of FIP1 and not the consequence of an off-target effect of any of the shRNAs, we expressed each of the FIP1 shRNAs constructs individually or in pair combinations together with adiponectin-myc in HEK293 cells and as above, adiponectin secretion was determined by ELISA. The result of these experiments is shown in Figure S2. Cells were first transfected with FIP shRNA constructs individually or in pair combinations or with all the shRNAs together (indicated as ‘shRNA pool’ in Figure S2) as an internal control. Cells incorporating the plasmids were selected in puromycin, and surviving cells were transfected with a construct expressing adiponectin-myc and allowed to recover for 24 hrs prior to measuring adiponectin secretion. We found that neither the expression of single nor double combinations of the FIP1 shRNA plasmids were able to increase adiponectin secretion above the amount seen in control cells expressing only the adiponectin-myc plasmid or adiponectin-myc and the shRNA empty vector (Figure S2 panel A). Importantly, neither single or double transfections of FIP1 shRNA constructs resulted in depletion of FIP1 protein expression (Figure S2 panel B), as this was only achieved when all the shRNAs were used in combination (Figure S2. panel B, lanes 11 and 12). These data confirmed that the effects seen on adiponectin secretion are not caused by an off-target effect of these shRNAs but rather the depletion of FIP1 expression when all the shRNAs are used in combination. In subsequent experiments we sought to confirm and further determine the function of FIP1 in adiponectin trafficking in adipose cells.

### 3.2: FIP1 depletion increases adiponectin secretion in differentiated adipocytes

**Figure 2:**
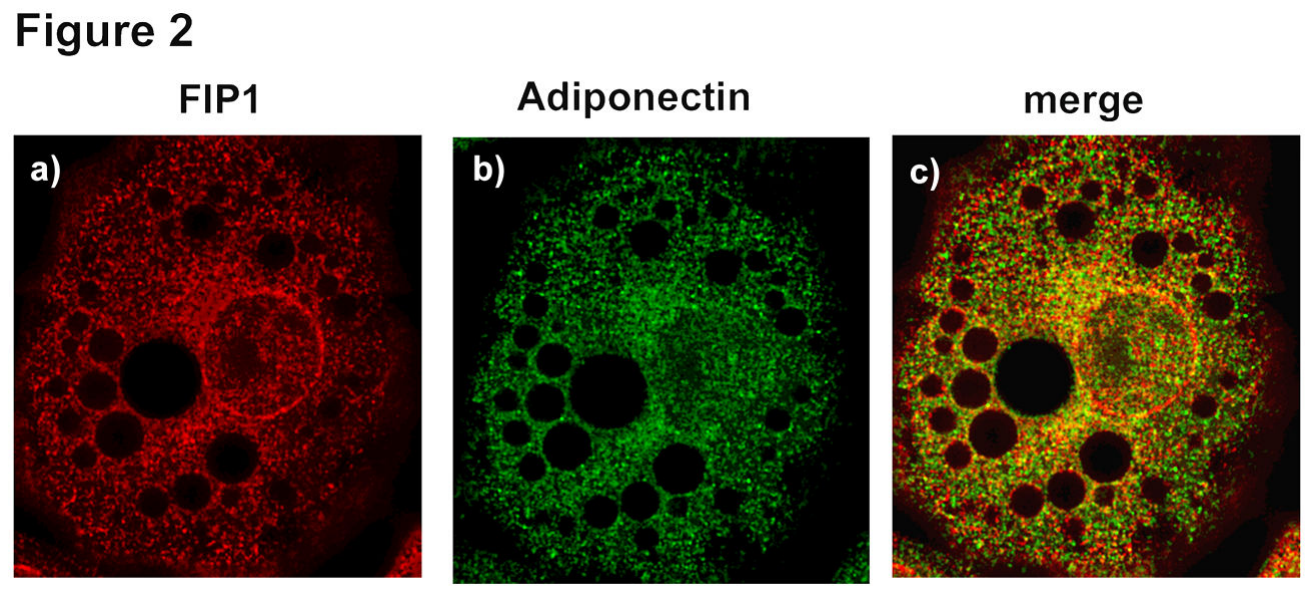
Intracellular distribution of FIP1, and adiponectin in adipocytes. 3T3L1 cells were cultured and differentiated to adipocytes as described in the methods section. Cells were fixed, permeabilized and immunostained with anti-FIP1 (Sigma) and anti-adiponectin (R and D) primary antibodies and Alexa-488 and Alexa-594 –conjugated secondary antibodies. Data shown is representative of cells obtained from three independent sets of cells. Determination of colocalization was carried out as described (n = 27).

To investigate whether FIP1 plays a role in adiponectin secretion in adipocyte cells, we first examined the intracellular distribution of FIP1 (rab11 FIP1C) in adipocytes. To this end, fully differentiated 3T3L1 adipocytes were fixed, permeabilized and immunostained with antibodies to detect endogenous FIP1 and adiponectin proteins. As shown in Figure 2 localization of FIP1 partially overlapped with that of endogenous adiponectin (Figure 2, panel c). Analysis revealed a high degree of FIP1 and adiponectin colocalisation (M1 0.99 ± 0.001, M2 0.95 ± 0.012, PC 0.67 ± 0.018).

**Figure 3:**
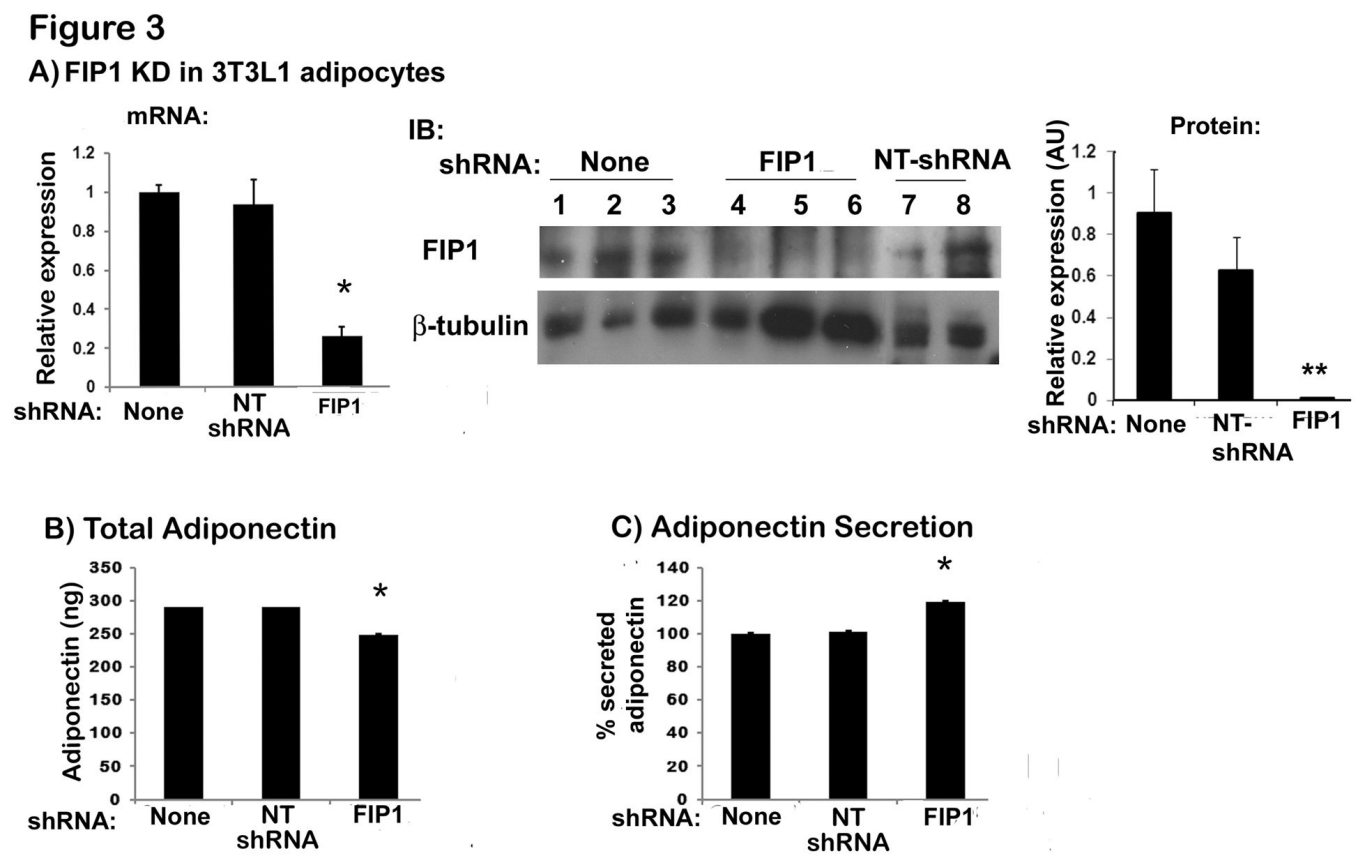
Knock down of FIP1 in 3T3L1 adipocytes increases adiponectin secretion. 3T3L1 cell lines stably expressing non-targeting shRNAs or shRNAs for FIP1 were generated as described in the methods section. Cells were cultured in the presence of 3-5 µg/ml puromycin to select for shRNA expressing cells and subsequently differentiated into adipocytes and used on day 10 after differentiation. **A**) Knock down of FIP1 in adipocytes. Left panel: total RNA was extracted from control (uninfected), non-targeting shRNA expressing and FIP1 shRNA expressing cells, and the mRNA for FIP1 was quantified by qPCR. Gene expression was normalized to Cyclophilin A using the ΔΔCT method. Graph is the mean ± SEM of 3 independent experiments each quantified in triplicate. Statistical analysis: one way ANOVA. *indicates p<0.05 relative to control cells. Middle panel: Cell lysates were obtained from differentiated control (non-infected) cells, cells expressing FIP1 specific shRNAs or non-targeting (NT) shRNAs. Samples were separated by SDS-PAGE, transferred to nitrocellulose filter and immunoblotted with an anti-FIP1 antibody or anti β-tubulin antibody as shown. The right panel shows quantification of the western blot normalized to tubulin. Statistical analysis: one way ANOVA, ** indicates p<0.01 relative to control non-infected cells. **B**) Adiponectin expression. Total intracellular adiponectin content was determined in control (non-infected) cells, in cells expressing non-targeting shRNAs or shRNAS for FIP1. Cellular lysates were obtained from fully differentiated cells, and adiponectin content quantified as described in the methods section. Data from two independent experiments, with n=5 replicates each. Data are mean ± SEM. * Statistic analysis: one way ANOVA, * indicates p<0.05 relative to control cells. **C**) Adiponectin secretion. Culture media and cell lysates were obtained from control cells (non infected), non-targeting shRNA expressing cells, or FIP1-depleted cells the amount of adiponectin quantitated using ELISA. Secretion was calculated as indicated in the methods section. Secretion is expressed as a percentage of total adiponectin expressed. Data from two independent experiments, with n=5 replicates each. Data are mean ± SEM. * indicates statistically different from control cells (p < 0.05). NT shRNA = non-targeting shRNA.

To examine whether FIP1 functions in adipocytes to regulate adiponectin secretion, we next generated stable adipose cell lines with depleted expression of FIP1 by constitutively expressing shRNAs for FIP1 or a non-targeting shRNA (NT shRNA) sequence as control. To achieve this, undifferentiated 3T3L1 cells were infected with a pool of lentiviral vectors each expressing a shRNA sequence for FIP1 or a non-targeting shRNA construct. As with the human FIP1 the constructs used for targeting FIP1 protein in mouse targeted all the isoforms of rab11 FIP1C. Infected cells were selected in the presence of puromycin and subsequently differentiated to obtain adipocyte cells. Expression of FIP1 was assessed by real time PCR analysis and western blot (Figure 3A). Expression of a non-targeting shRNA did not reduce the mRNA levels of FIP1 in adipocytes, however mRNA levels for FIP1 were reduced by 80% in the FIP1 shRNA expressing cells compared to control non-infected (Figure 3A, left panel). At the protein level, FIP1 expression was reduced by 96% in shRNA expressing cells (Figure 3A, middle and right panels) compared to non-infected adipocytes. We then examined the total amount and the secretion of endogenous adiponectin in uninfected cells, in cells expressing non-targeting shRNAs or FIP1 shRNAs. The expression of non-targeting shRNAs did not alter adiponectin content or secretion. In contrast, adipocytes expressing FIP1 shRNAs displayed a small (16%) but significant decrease in the total amount of adiponectin (Figure 3B), yet, despite this, these cells exhibited an increase in adiponectin secretion of approximately 20% (Figure 3C). Taken together with the data from HEK293 cells, these results suggest that FIP1 is involved in regulating adiponectin trafficking and secretion in adipocytes.

**Figure 4:**
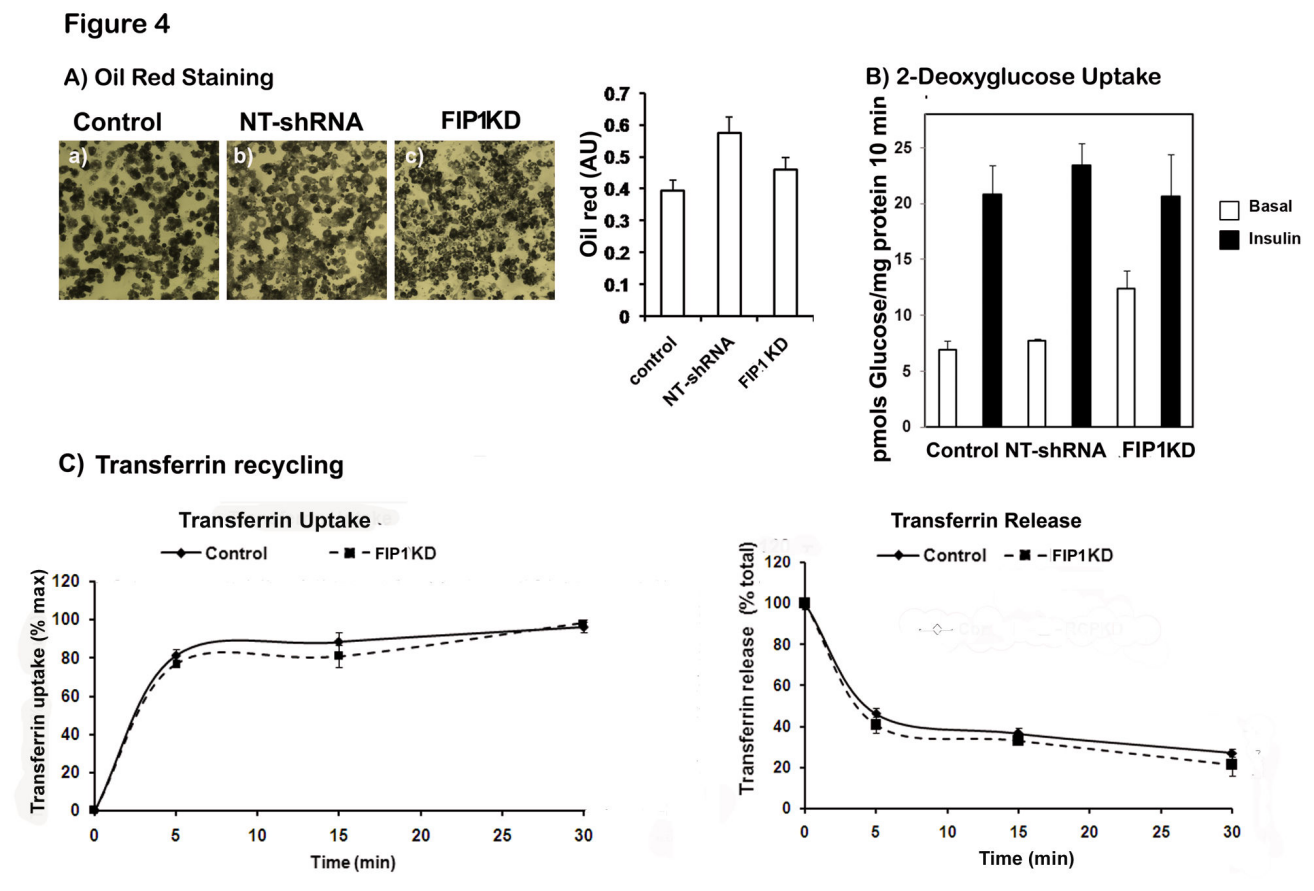
Knock down of FIP1 does not alter adipogenesis, insulin-stimulated Glut4 translocation or transferrin receptor recycling. Control 3T3L1 cells (non-infected), and 3T3L1 cell lines stably expressing a non-targeting shRNA or shRNAs for FIP1 were cultured and differentiated to adipocytes and used on day 10 after differentiation. **A**) Cells were fixed and stained with oil red and visualized under a light microscope. Images were taken in a Leica microscope. Representative images are shown from at least three independent experiments. Oil red stain was extracted in isopropanol and the absorbance quantified at 500 nm. **B**) Cells were serum starved in DMEM for 3 hours and either left untreated or treated with insulin (100 nM) for 30 minutes. ^3^H-2-deoxyglucose uptake was then measured for 10 minutes as indicated in the material and methods section. ^3^H -2-deoxyglucose uptake at 1 mM final concentration for 10 minutes is within the linear range in these cells (not shown) **C**) Transferrin recycling. Transferrin uptake: control and FIP1 Knock down 3T3L1 cells were incubated with Alexa-488 conjugated transferrin as indicated in the methods section. At different time points cells were washed and the intracellular fluorescence determined by flow cytometry. Transferrin release: Cells were first loaded with Alexa-488 transferrin for 45 minutes at 37^°^C and then washed at different time points as indicated. Cells were collected and intracellular fluorescence quantitated by flow cytometry as described in the methods section. Results show mean ± SEM of 2 independent experiments quantified in triplicate.

To confirm that the changes seen in adiponectin synthesis and secretion observed in FIP1 knock down cells were not due to alterations in the differentiation of the cells, we examined lipid accumulation by oil red staining and the expression and recruitment to the plasma membrane of the facilitative glucose transporter Glut4 in response to insulin. FIP1 depleted cells exhibited a similar differentiation to control cells as judged by phase microscopy and lipid accumulation (Figure 4A). These cells exhibited comparable expression levels of the glucose transporter Glut4 (not shown) and responded to insulin treatment in a comparable manner to control cells, by redistributing the glucose transporter Glut4 to the plasma membrane and stimulating glucose uptake in response to insulin (Figure 4B). These findings suggest that adipocyte differentiation and Glut4 traffic are not affected by FIP1 knock down.

To further evaluate the role of FIP1 in regulating adipocyte endosomal trafficking we assessed recycling of transferrin receptors in control uninfected cells or FIP1 depleted cells. To this end, cells were incubated with Alexa488-conjugated transferrin on ice for 10 minutes. Transferrin occupied receptors were then allowed to internalize at 37^°^C for different lengths of time ranging from 0 to 30 minutes. At each time point, cells were washed and the amount of intracellular fluorescence quantified by flow cytometry. As seen in Figure 4C, we did not observe any differences in the transferrin uptake rates in cells depleted of FIP1 compared to control cells. We then determined the exocytosis of transferrin receptors following a pre-loading with Alexa-488 transferrin for 30 minutes at 37^°^C. Transferrin release was carried out at 37^°^C, at different time points cells underwent washes as described in the methods, and the intracellular accumulation of fluorescently labeled transferrin determined by flow cytometry. We found that the release of transferrin was no different in FIP1 knock down cells compared to control cells (Figure 4C). These findings suggest that FIP1 knock down does not alter the cycling of transferrin receptors in adipocytes.

### 3.3: FIP1 Knock down does not impair trafficking of adiponectin to the Early/Sorting endosome (EE/SE) or recycling endosome (ERC)

**Figure 5:**
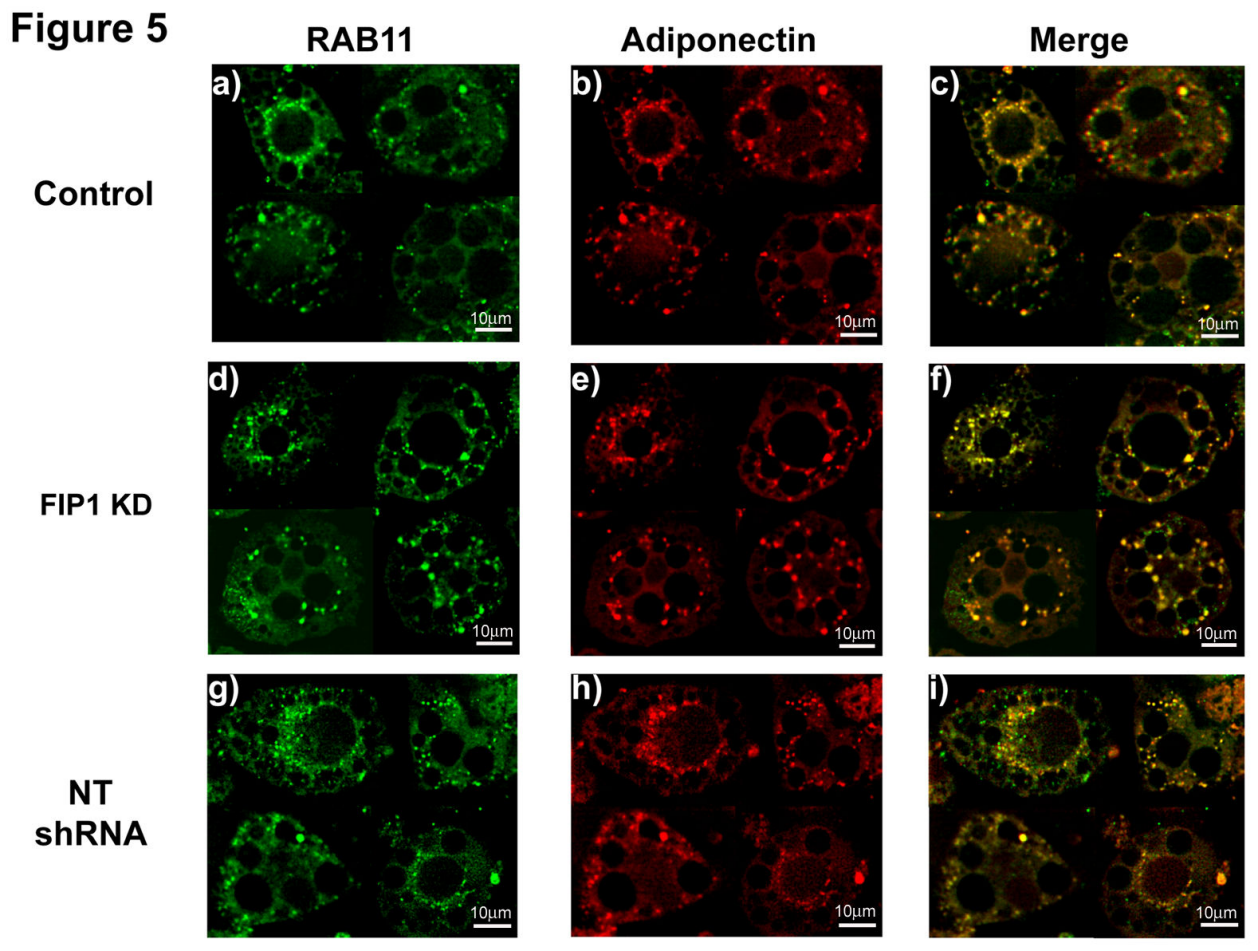
Knock Down of FIP1 does not impair traffic of adiponectin to the ERC compartment. Control uninfected 3T3L1 cells or expressing non-targeting shRNAs or FIP1- shRNAs cultured and differentiated to adipocytes and used on day 10 after differentiation. Cells were fixed, permeabilized and stained with specific antibodies to detect endogenous rab11 and adiponectin proteins. Secondary antibodies conjugated to Alexa-488 or Alexa-594 respectively were used to visualize the proteins. Cells were visualized using a Leica DMI6000 microscope. Representative cells from 3 independent experiments are shown. Determination of colocalization was carried out as described (Control (n = 10), FIP1 shRNA (n = 9), NT shRNA (n = 4)). Panels c, f and i show the merged images.

To shed some light on the mechanism of action of FIP1 and to rule out that the increase in secretion of adiponectin is due to mistargeting of intracellular adiponectin in the FIP1 depleted cells, we next examined the intracellular compartmentalization of adiponectin in the FIP1 knock down adipocytes, and compared it to that of non-infected control cells or cells expressing the non-targeting shRNAs. First, we determined the distribution of endogenous adiponectin and rab11. We found that intracellular adiponectin co-distributed with rab11 in control cells (M1 0.96 ± 0.02, M2 0.95 ± 0.01, PC 0.81 ± 0.02), in cells expressing the non-targeting shRNA construct (M1 0.96 ± 0.02, M2 0.99 ± 0.001, PC 0.80 ± 0.04) and in FIP1 knock down cells (M1 0.98 ± 0.01, M2 0.98 ± 0.01, PC 0.87 ± 0.03) (Figure 5, merged images, panels c, f, and i). Statistical analysis (ANOVA) revealed no differences in the colocalization levels of adiponectin and Rab11 between control, non-targeting shRNAs and FIP1 depleted cells (p > 0.05). These results demonstrate that FIP1 depletion does not prevent traffic of endogenous adiponectin to the ERC.

It has been reported that in addition to rab11 FIP1 can also interact with rab14 [23]. We examined whether endogenous adiponectin distributed with intracellular rab14 in control or FIP1 depleted adipocytes. We found that endongenous adiponectin partially overlapped with that of rab14 in differentiated 3T3L1 adipocytes (Figure S3), but no differences (p > 0.05) were seen between control (M1 0.72 ± 0.09, M2 0.98 ± 0.01, PC 0.55 ± 0.07) or FIP1 depleted adipocytes (M1 0.73 ± 0.10, M2 0.97 ± 0.01, PC 0.53 ± 0.10).

**Figure 6:**
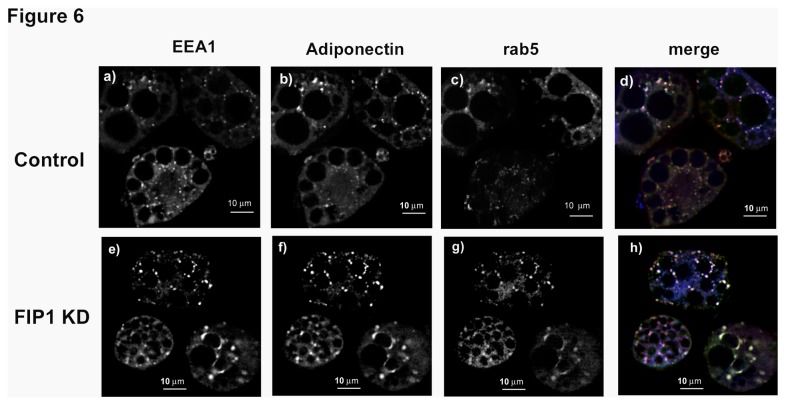
Knock down of FIP1 does not impair traffic of adiponectin to the sorting endosome. Control, uninfected 3T3L1 cells, or cells stably expressing shRNAs for FIP1 were cultured and differentiated to adipocytes and used on day 10 after differentiation. Cells were fixed, permeabilized and stained with specific antibodies to detect endogenous EEA1, Rab5 and adiponectin proteins. Secondary antibodies conjugated to Alexa-350, Alexa-488 and Alexa-594 were used to visualize the proteins. Cells were imaged in a Leica DMI6000 microscope. Representative cells are shown obtained in three independent experiments. Determination of colocalization was carried out as described (Rab 5: Control (n = 9), FIP1 shRNA (n = 7); EEA1: Control (n = 10), FIP1 shRNA (n = 11) Panels d and h show the merged images.

Since previous observations also suggested that adiponectin partially colocalized with rab5 in the early/sorting endosome (EE/SE) [15], we next compared the distribution of adiponectin with markers for the EE/SE (rab5 and EEA1 –early endosome antigen1-) in control or FIP1 knock down adipocytes. In control cells adiponectin distribution overlapped with that of rab5 (M1 0.97 ± 0.02, M2 0.89 ± 0.02, PC 0.67 ± 0.03) and EEA1 proteins (M1 0.91 ± 0.03, M2 0.99 ± 0.01, PC 0.61 ± 0.07) (Figure 6, panel d). This distribution was maintained in the FIP1 knock down cells (rab 5: M1 0.99 ± 0.0002, M2 0.92 ± 0.0019, PC 0.85 ± 0.022; EEA1: M1 0.96 ± 0.01, M2 0.94 ± 0.06, PC 0.61 ± 0.06) (Figure 6, panel h). These results demonstrate that FIP1 depletion does not prevent traffic of endogenous adiponectin to the early endosome.

**Figure 7:**
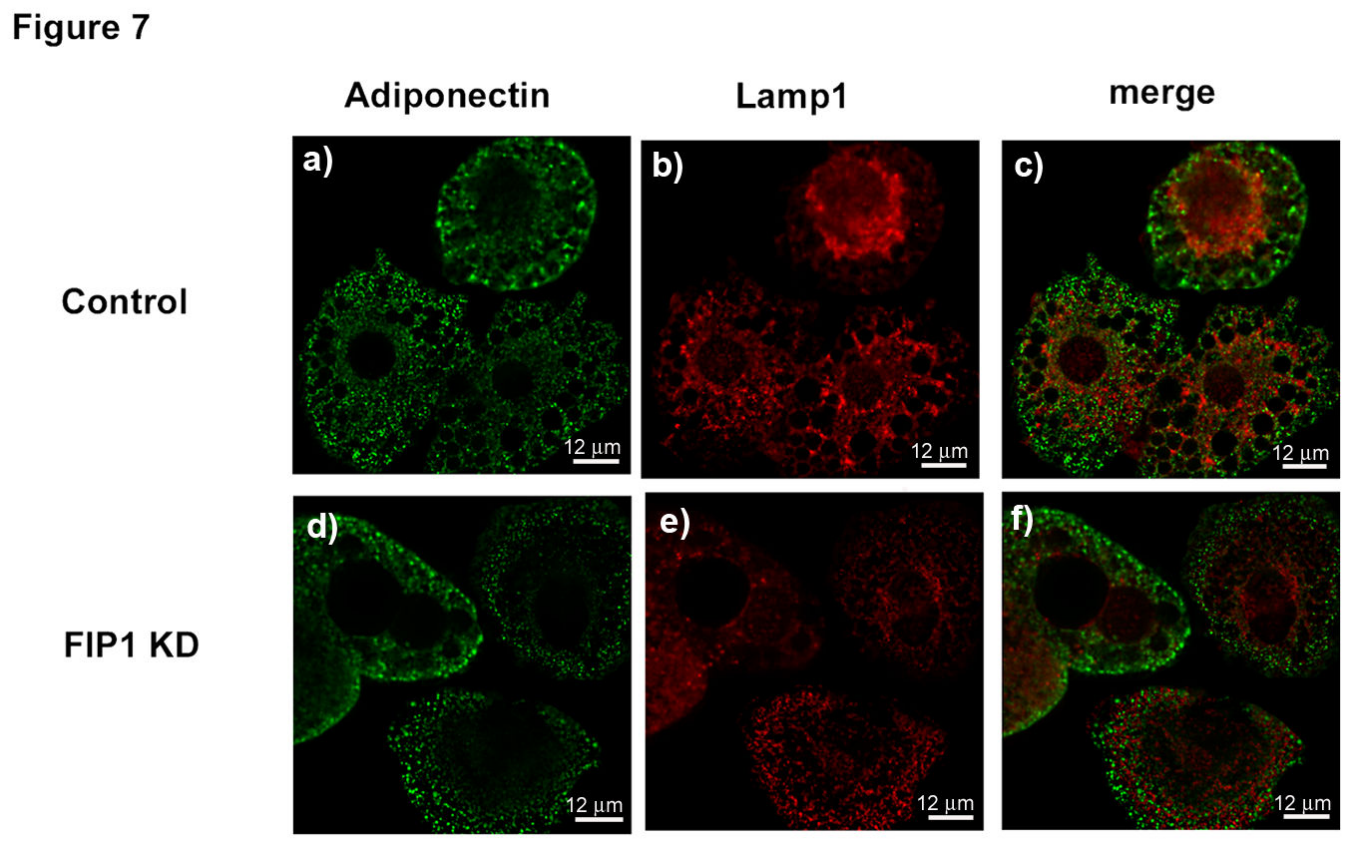
Adiponectin does not localize with Lamp1 containing membranes in adipocytes. Control (uninfected) 3T3L1 cells or cells expressing shRNAs for FIP1 were cultured and differentiated to adipocytes and used on day 10 after differentiation. Cells were fixed, permeabilized and stained with specific antibodies to detect endogenous adiponectin or lamp1. Secondary antibodies conjugated to Alexa488 and Alexa594 were used to visualize the proteins. Representative cells are shown from 3 independent experiments. Panels c, and f show the merged images.

Since cargo from the ERC/EE can be delivered to the late endosome (LE) for degradation we next evaluated the possibility that FIP1 may be regulating traffic of adiponectin to the late endosome. To this end, we evaluated the intracellular distribution of adiponectin and Lamp1, a marker for the late endosome, in control or FIP1 depleted adipocytes. As shown in Figure 7 we found that intracellular distribution of adiponectin was not overlapping with that of Lamp1 (Figure 7, merged panels c and f). These findings suggest that adiponectin does not traffic from the EE/ERC to the late endosome.

### 3.4. FIP1 knockdown does not impair internalization of adiponectin receptors

**Figure 8:**
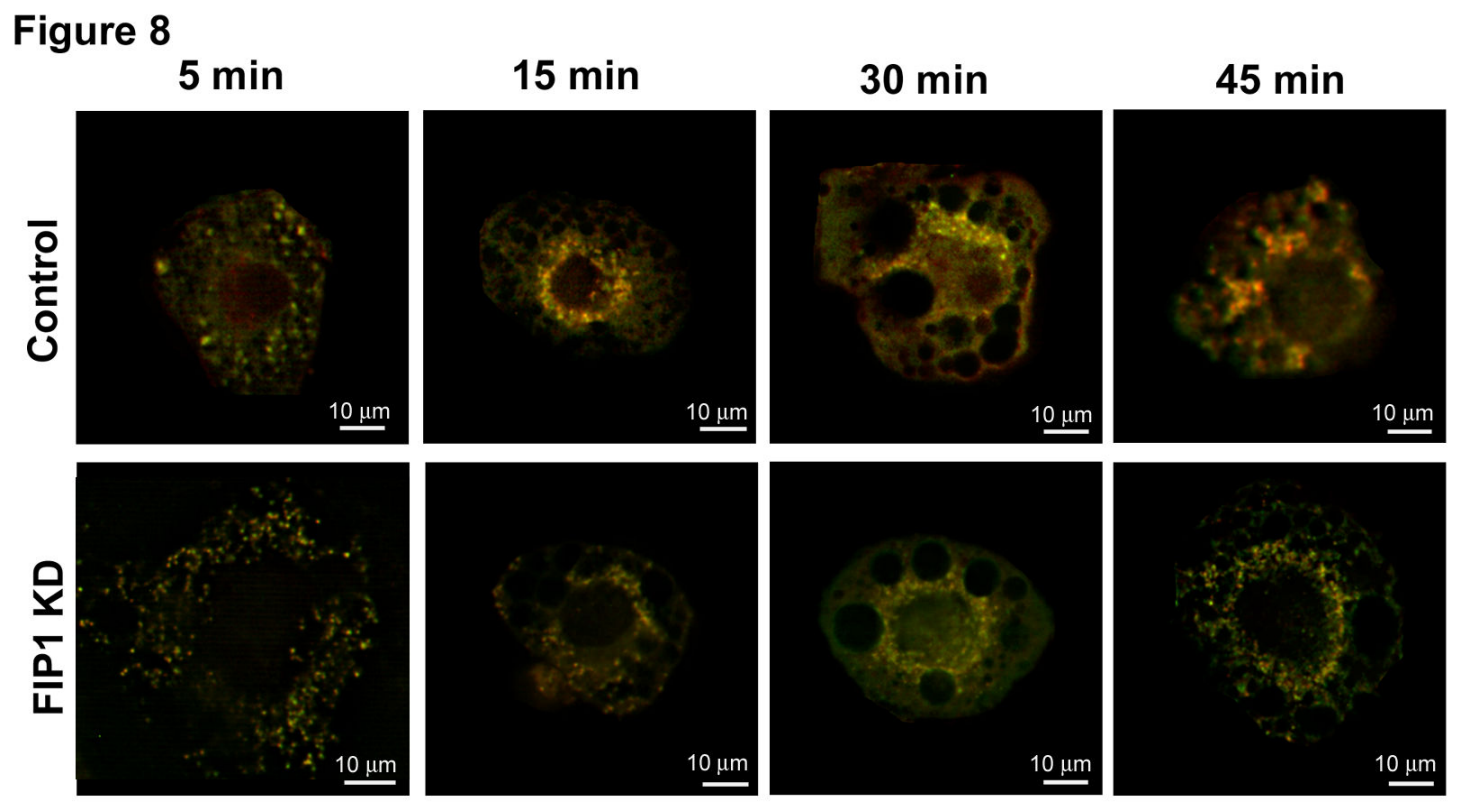
Adiponectin receptors are internalized and localize with transferrin receptor in intracellular membranes. Control uninfected 3T3L1 cells or cells expressing shRNAs for FIP1 were cultured and differentiated to adipocytes and used on day 10 after differentiation. Cells were labelled with biotin-adiponectin and Alexa-594 conjugated transferrin at 4^°^C for 30 minutes and then incubated in Alexa488-streptavidin. Internalization of adiponectin and transferrin bound receptors was allowed at 37^°^C for the times as indicated. Cells were then transferred to ice, and fixed in PFA as detailed in the methods section. Cells were imaged in a Leica DMI6000 microscope. Data shown are the merged images. Representative cells are shown selected from three independent experiments.

Since adipocytes express high levels of adiponectin receptors, and since a fraction of released adiponectin can be internalized through binding to these receptors, we tested the possibility that FIP1 knock down could prevent internalization of adiponectin receptors. To this end we labeled adiponectin receptors with biotinylated adiponectin and used Alexa-592 conjugated transferrin to monitor internalization of transferrin receptors as control. Cells were incubated with the ligands on ice for 30 minutes followed by incubation with streptavidin-conjugated Alexa-488. Cells were then transferred to 37^°^C and the adiponectin and transferrin occupied receptors were allowed to internalize for different lengths of time ranging from 5 to 45 minutes. Cells were then washed in PBS, fixed and imaged on an epifluorescent microscope. Our results demonstrate that FIP1 knock down cells display substantial expression of adiponectin receptors and that these internalize and co-distribute intracellularly with transferrin receptors (Figure 8). No differences were observed in the intracellular distribution of adiponectin receptors between control cells and FIP1 depleted cells (Figure 8).

### 3.5: FIP1 expression is downregulated during adipogenesis and by treatment with thiazolidinediones

Adiponectin levels increase during adipocyte differentiation and by treatment with the PPARγ agonists thiazolidinediones [31] [32]. To further assess the physiological relevance of FIP1 function we sought to determine whether FIP1 expression parallels with that of adiponectin in these conditions.

**Figure 9:**
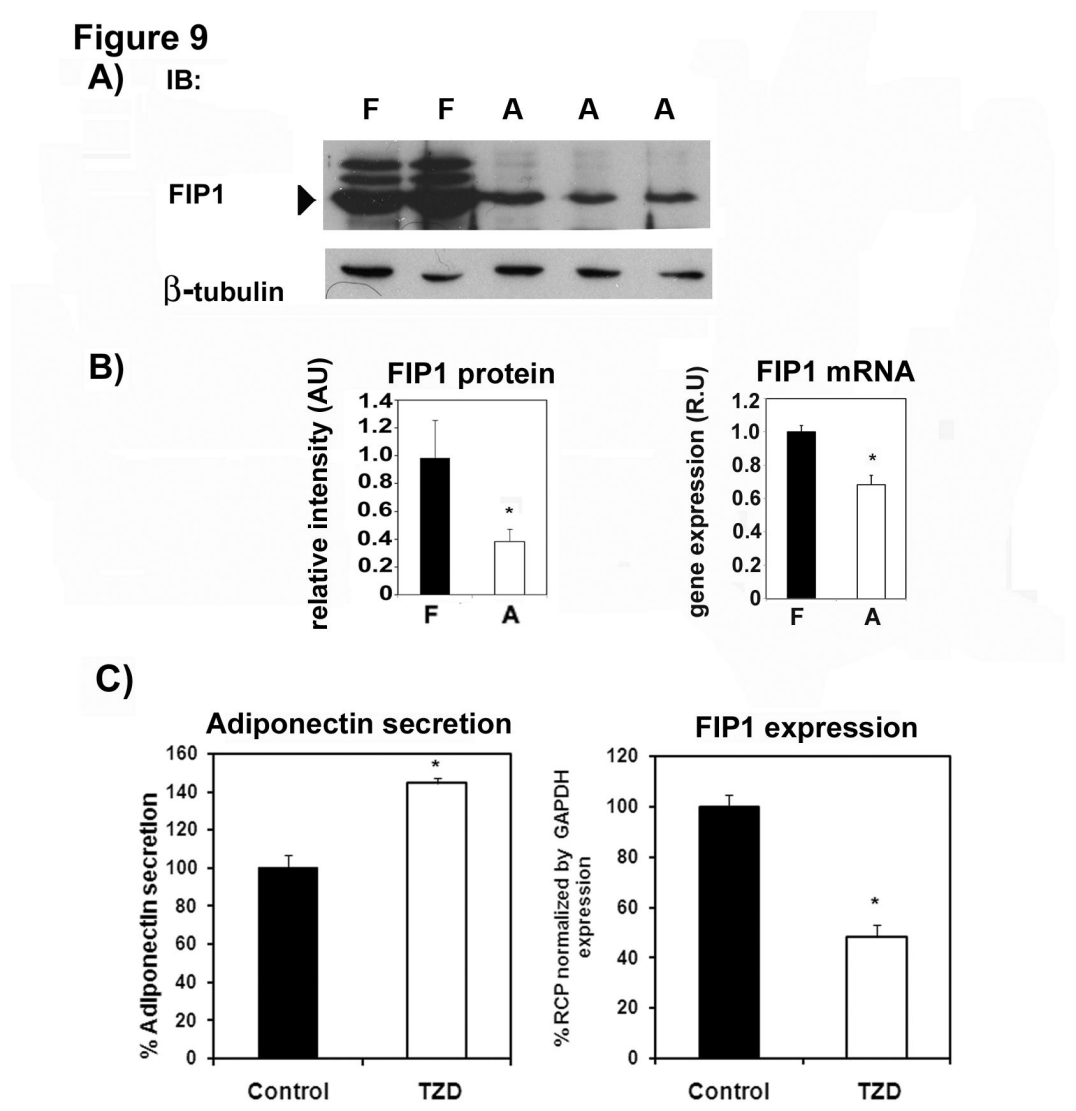
FIP1 expression decreases during adipogenesis, and by treatment with troglitazone. **A**) Cell lysates were obtained from 3T3L1 fibroblasts (F) or fully differentiated adipocytes (A). Samples were separated by SDS-PAGE, transferred to a nitrocellulose filter and immunoblotted with an anti-FIP1 or β-tubulin antibody as shown. Representative blot of two independent experiments. **B**) Quantification of FIP1 protein levels (left panel) or FIP1 mRNA levels (right panel) following the differentiation of 3T3L1 cells to adipocytes. Data shows mean ± SEM of triplicate values. Statistic analysis: One way ANOVA * indicates p<0.05. **C**) 3T3L1 cells were cultured and differentiated to adipocytes. At day 8 of differentiation, cells were either left untreated or treated with vehicle or with 10 µM troglitazone for 3 days. Conditioned media and cellular lysates were harvested for adiponectin quantification by ELISA as described in the methods section A subset of cell plates were used for total RNA isolation and mRNA for FIP1 was quantified by real time PCR as described in the methods section. Data shows mean ± SEM of 2 independent experiments each performed in triplicate. Statistical analysis, one way ANOVA: * indicates p<0.05.

To this end we first examined the expression of FIP1 in undifferentiated and differentiated 3T3L1 cells. We found that FIP1 protein (Figure 9A and B) and mRNA levels (Figure 9, panel B) decreased with the differentiation of 3T3L1 cells to adipocytes.

In order to confirm these data, we treated fully differentiated adipocytes with troglitazone, a thiazolidinedione that functions as a Peroxisome Proliferator-Activated Receptor gamma (PPARγ) agonist. PPARγ together with the transcription factor C/EBPs (CCAAT/enhancer-binding proteins) play a crucial role in regulating the differentiation programme in adipocytes regulating many lipid and glucose homeostatic genes. Since thiazolidinediones potentiate adipocyte differentiation we postulated that troglitazone would reduce the expression of FIP1 in adipocytes. Treatment of fully differentiated adipocytes with troglitazone increased adiponectin secretion (Figure 9C left panel) and as expected reduced the expression of FIP1 (Figure 9C, right panel). Taken together these data suggest that FIP1 protein is reduced during adipocyte cell differentiation possibly via the activation of PPARγ.

### 3.6: FIP1 expression inversely correlates with BMI and obesity markers in humans

Since adiponectin levels decrease in obesity and type 2 diabetes [33], we sought to determine whether FIP1 expression in adipose tissue is altered in obesity in humans. To this end, we analyzed 233 adipose tissue samples, 123 visceral (VAT) and 110 subcutaneous (SAT), obtained from subjects with a body mass index (BMI) ranging between 20 and 58 kg/m^2^. We correlated the gene expression of FIP1 with obesity markers and with several genes involved in lipid metabolism and insulin signaling.

**Figure 10:**
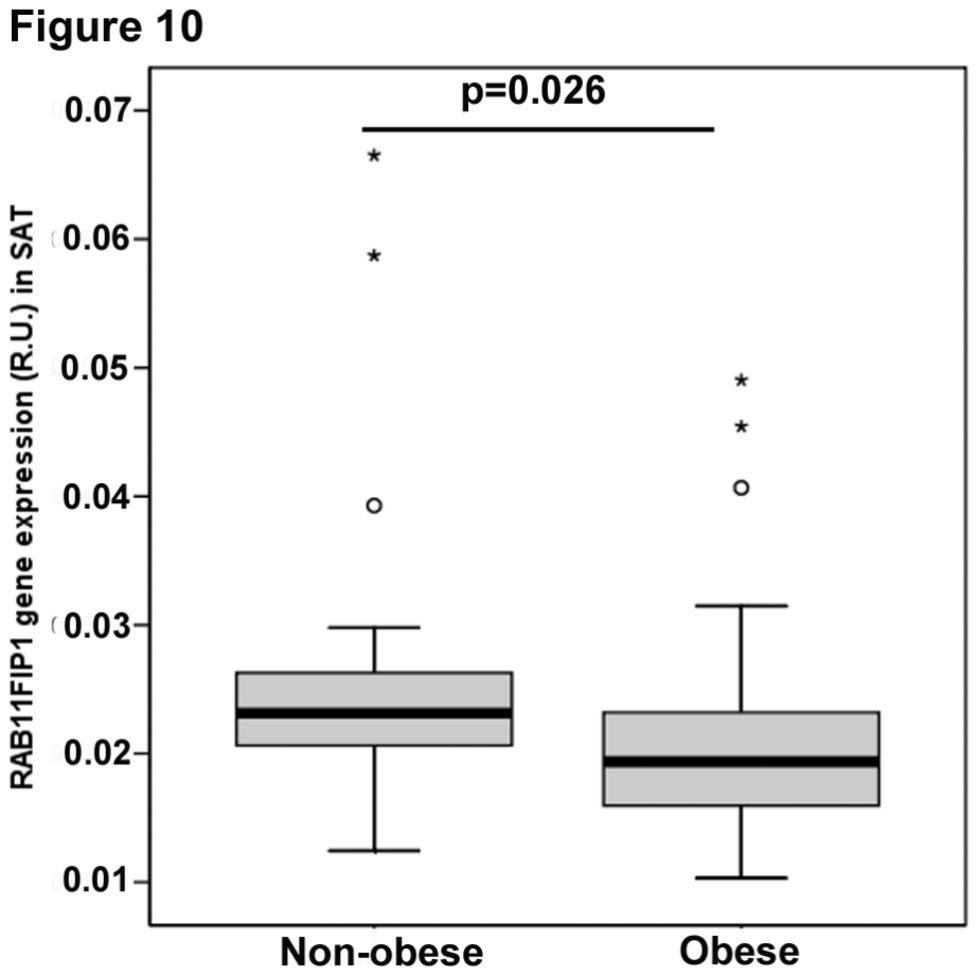
Expression of FIP1 mRNA in subcutaneous adipose tissue in association with obesity status. Expression of FIP1 gene in human adipose tissue biopsies was quantified as described in the methods section and correlated with BMI (body mass index). Subjects were classified as non-obese if BMI<30 kg/m^2^(N=21) and obese if BMI>30 kg/m^2^(N=55). Diabetic patients were excluded. Boxplots showing the median, interquartile range, outliers, and extreme cases. Statistical analysis T-test, p=0.026.

Interestingly we found that gene expression of FIP1 was significantly increased in visceral compared with subcutaneous adipose tissue (0.029 ± 0.009 vs. 0.023 ± 0.008, p<0.0001). We also found that FIP1 expression inversely correlated with triglyceride levels and positively correlated with mRNA levels of insulin receptor substrate-1, a protein involved in insulin signaling in both SAT and VAT (Table 1). In SAT there was an inverse relationship between both BMI and mRNA leptin levels and FIP1 gene expression, however, these associations were not significant in VAT (Table 1). When subjects were classified according to their BMI, significant reductions in FIP1 expression were seen in obese (BMI>30 kg/m^2^) subjects (Figure 10) when compared with lean or non-obese individuals. Taken together these data support our *in vitro* observations in that less FIP1 expression is found in more mature adipocytes that accumulate more lipids, and highlight the physiological importance of the regulation of FIP1 expression in adipocytes.

**Table 1:** Bivariate correlation between sex-adjusted RAB11FIP1 gene expression, antropometric and clinical parameters, and expression of selected genes.

	**SAT**		**VAT**	
	r	p	r	p
Age (years)	-0.083	0.5	0.05	0.6
BMI (kg/m2)	-0.22	**0.03**	-0.11	0.25
Fasting glucose (mmol/l)	-0.01	0.8	-0.035	0.7
HbA1c (%)	0.08	0.5	0.04	0.07
HDL-Cholesterol (mg/dl)	0.20	**0.04**	0.11	0.4
Fasting triglycerides (mg/dl)	-0.03	0.7	-0.22	**0.03**
PPARγ (R.U.)	0.11	0.37	0.12	0.36
IRS1 (R.U.)	0.33	**0.005**	0.24	**0.03**
LEP (R.U.)	-0.34	**0.005**	-0.15	0.2
FASN (R.U.)	0.24	**0.03**	0.10	0.4

Sample size: N=123 (VAT) and N=110(SAT)

R.U.: relative units of gene expressionSAT: Subcutaneous adipose tissue; VAT: Visceral adipose tissue

### 3.7. FIP1 expression increases with exposure to the pro-inflammatory cytokine tumor necrosis factor alpha (TNFα).

**Figure 11:**
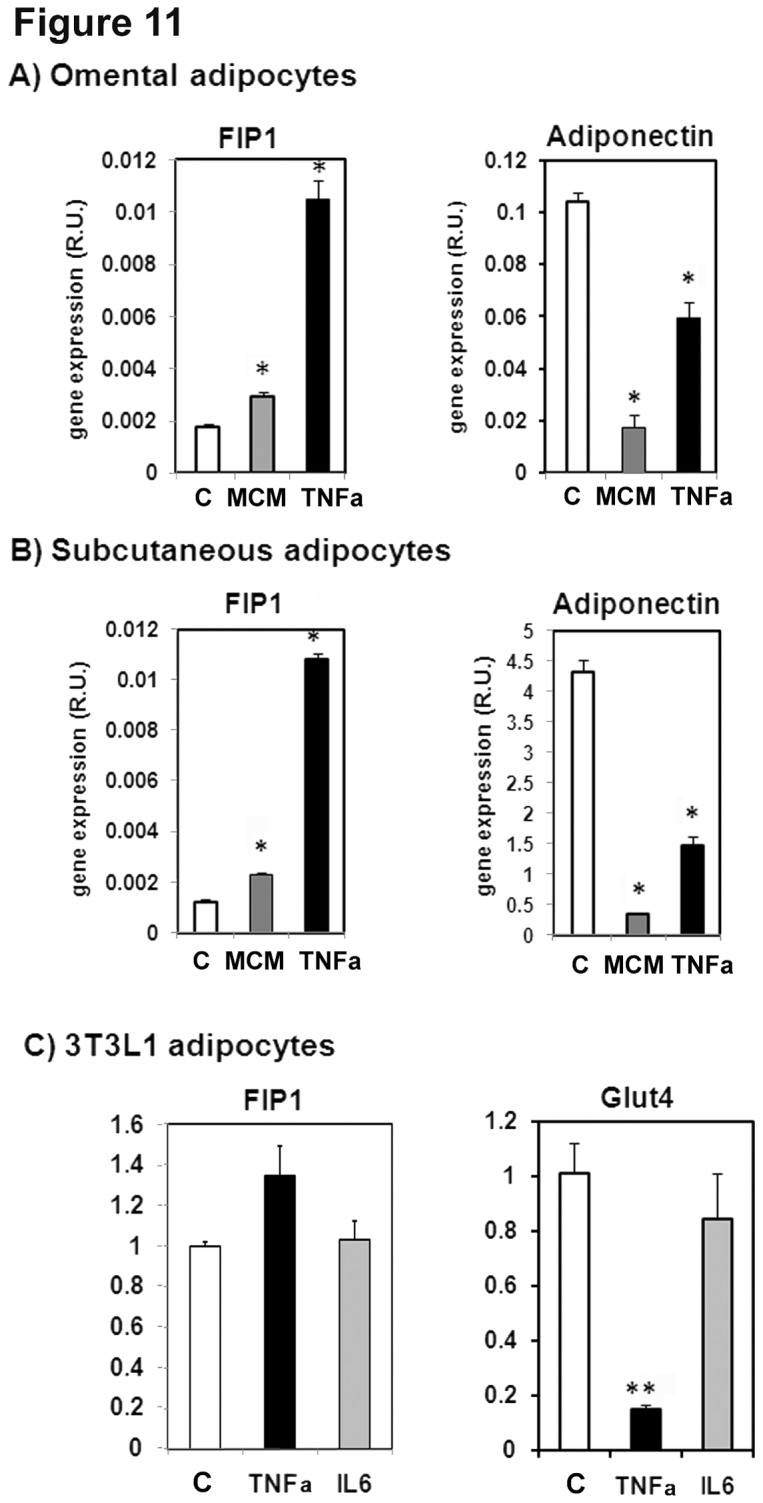
Pro-inflammatory cytokines increase the expression of FIP1 in adipocytes. Human preadipocyte cells from omental (**A**) or subcutaneous (**B**) adipose tissue were obtained from Zen-Bio and differentiated as per their instructions for 14 days. A subset of cells were left untreated (control, C) or were incubated in media supplemented with macrophage conditioned media (MCM, 5% v/v) or with 100 ng/ml of Tumor necrosis factor alpha (TNFα), Total RNA was isolated and FIP1 and adiponectin mRNA was quantitated by real time PCR as indicated in the methods section. Relative quantification was carried out using the ∆∆Ct method using PPIA gene expression as an internal control. Statistical analysis: One way ANOVA. * indicates statistical significance p<0.05. Data from one experiment with triplicate cell dishes. **C**) Fully differentiated 3T3L1 adipocytes were incubated in the absence (control, C) or presence of TNFα (TNFα) or Interleukin 6 (IL6) at 50 ng/ml for 24 hours, Total RNA was isolated and the amount of mRNA for FIP1, or Glut4 was determined by real time PCR. Quantification was carried out using the ∆∆Ct method using cyclophyllin A as internal control. The graph shows data from a representative experiment of two independent experiments each carried out with triplicate cell dishes for each group. Statistical analysis: One way ANOVA, ** indicates p<0.01.

To gain further insights into the regulation of FIP1 expression in adipose tissue and during adipose tissue dysfunction as it occurs in obesity we cultured human adipose cells in the absence or presence of the pro-inflammatory cytokine TNFα or in media supplemented with 5% (v/v) of macrophage conditioned media obtained after stimulating THP macrophages with LPS for 24hrs. As seen in Figure 11, exposure of differentiated adipocytes from visceral (panel A) or subcutaneous origin (panel B), to either TNFα or to macrophage conditioned media resulted in a significant increase in FIP1 expression. Concomitantly with this, and consistent with literature findings [34], adiponectin mRNA levels were decreased by exposure to pro-inflammatory cytokines (Figure 11 A and B right panels). We confirmed these findings in the mouse cell line 3T3L1, by incubating differentiated 3T3L1 adipocytes in the presence of TNFα or interleukin-6 (IL-6) for 24 hrs. As control for this experiment we determined the mRNA levels of the glucose transporter Glut4, which has been reported to decrease with exposure to TNFα [34,35] or to IL-6 [36]. We found that TNFα increased FIP1 mRNA levels in 3T3L1 adipocytes while profoundly decreased Glut4 mRNA levels (Figure 11C). No significant changes were observed in adipocytes treated with IL-6 (Figure 11C).

Hypoxia has been reported to be a factor regulating adipokine and cytokine production by adipose tissue in obesity. Therefore to further confirm these findings we determined the expression of mRNA for FIP1 in cells cultured under hypoxic conditions (1% Oxygen, 5% CO_2_ and 94% N_2_) for 8, 16 or 24 hours and compared it to that of cells cultured under normoxic conditions (5% CO_2_, 95% air). As positive control for the hypoxia treatment we measured the mRNA abundance of the glucose transporter Glut1 which is known to be increased under hypoxic conditions. As expected, hypoxia raised the mRNA levels of Glut1 (Figure S4, panel B). There was a tendency for the cells exposed to hypoxic conditions to have higher FIP1 mRNA levels compared to normoxic cells but the differences did not reach statistical significance (Figure S4, panel A).

Taken together these findings indicate that TNFα contributes to regulate FIP expression in adipocytes.

## Discussion

We have previously reported that endosomes constitute an important compartment for the regulation of adiponectin secretion in cultured and rat isolated adipocytes. We previously described that intracellular adiponectin partially colocalizes with both rab5- and rab11-containing endosomes and either expression of rab5 or rab11 mutants, or chemical ablation of the endosomes, significantly reduces adiponectin release in adipocytes [15]. Here we set out to investigate the role of the rab11 family of interacting proteins (FIPs) in adiponectin trafficking and secretion.

Overexpression of FIP1, FIP3 or FIP5 in HEK293 cells inhibited adiponectin release whereas expression of FIP2 or FIP4 had no effect. Therefore, it is unlikely that FIP2 or FIP4 play a role in adiponectin secretion. While FIP3 overexpression in HEK293 cells resulted in a decrease of approximately 40% in adiponectin secretion, FIP3 depletion had no effect. Recent findings by Horgan et al., indicate that FIP3 overexpression fragments the Golgi compartment [37], and thus, it is likely that the decrease in adiponectin secretion observed by the overexpression of FIP3 is caused indirectly by the disruption of the Golgi apparatus. The lack of effect observed in the knock down condition suggests FIP3 does not play a role in adiponectin secretion and supports this theory. In our study, FIP5 overexpression in HEK293 cells resulted in a modest decrease in adiponectin secretion of approximately 35%, whereas FIP5 knock down decreased secretion by approximately 25%. However, much bigger effects were seen with FIP1. Overexpression of FIP1 in HEK293 cells decreased adiponectin release by approximately 55%, whereas knock down increased secretion by 70%. In 3T3L1 adipocytes intracellular distribution of FIP1 overlapped with that of endogenous adiponectin and crucially the effect of FIP1 depletion on adiponectin secretion was maintained albeit to a lesser degree (~20% increased secretion). Importantly however, the expression of FIP1 is severely downregulated during adipogenesis, thus the reduced effect of FIP1 knock down may be explained by the already reduced levels of FIP1 protein in adipocytes.

Concomitantly with increased adiponectin secretion in FIP1 depleted adipocytes we observed a small (~16%) but significant decrease in total adiponectin levels. As we find greater adiponectin secretion in this model, this may lead to a reduction in intracellular adiponectin. Adiponectin can also regulate its own production via binding to its receptor in adipocytes [38]. Furthermore, recent findings also suggest that adiponectin can modulate the expression of other adipokines in adipocytes, which in turn, could have an effect on adiponectin expression [39].

We found that FIP1 knock down in adipocytes increased adiponectin release without affecting the delivery of intracellular adiponectin from the trans-Golgi network to the SE/EE, or to the ERC. FIP1 knock down did not affect transferrin receptor recycling or exocytosis of the Glut4 glucose transporter in response to insulin. Our findings agree with those reported by Peden et al. [40], who found that FIP1 knock down in HeLa cells did not alter transferrin recycling. These authors however, found reduced levels of transferrin receptors in FIP1 knock down cells, which was prevented by lysosomal protease inhibitors, indicating that FIP1 could have a role in the sorting of transferrin receptors at the ERC away from the degradative pathway. While we did not quantify transferrin receptor expression in our adipocytes, our immunofluorescence experiments failed to detect any colocalization of adiponectin with Lamp1, a marker for the late endosome, in FIP1 depleted cells or indeed in control cells, suggesting adiponectin is not delivered to the late endosome. We found that adiponectin receptors internalize with transferrin receptors in adipocytes but there were no differences in the intracellular distribution of adiponectin receptors between control or FIP1 knock down cells. Furthermore, no differences were observed in transferrin receptor recycling rates. Taken together these data suggest that FIP1 is not regulating endocytosis of adiponectin receptors. Recently, Jing et al. [41], have reported a role for FIP1 in regulating traffic of selected cargo from the ERC to the TGN. Thus, it is also possible that the retention of adiponectin by FIP1 in adipocytes may involve the TGN. In any case, our secretion data obtained both in HEK293 and adipocytes is consistent with a role for FIP1 in the intracellular retention of adiponectin.

FIP1 has been shown to bind to both rab11 and rab14 [23]. We found that the distribution of endogenous adiponectin partially overlaps with that of rab14 in adipocytes. This suggests that the actions of FIP1 in regulating adiponectin secretion may be in part carried out through the interaction of FIP1 with rab14. However, further experiments are needed to evaluate the specific contributions of rab14 to adiponectin secretion which we plan to follow up in future studies.

Adiponectin is only expressed in fully differentiated adipocytes. Consistent with the above role of FIP1 in regulating adiponectin trafficking and secretion we found that FIP1 mRNA and protein levels are downregulated during adipocyte differentiation. Two families of transcription factors are key regulators of the adipogenic programme: the CCAAT/enhancer-binding proteins (C/EBPs) and the peroxisome proliferator-activated receptors (PPARγ). Treatment of differentiated adipocytes with the thiazolidinedione (TZD), troglitazone, a PPARγ agonist also reduced FIP1 levels while increased adiponectin secretion. While similar effects of TZDs on adiponectin secretion had been reported previously [32,42,43], the contribution of TZDs to regulating membrane traffic events in adipocytes have not been extensively studied. In a recent study, Martinez et al. 2010 [44] found that rosiglitazone, another TZD, enhanced endosomal traffic and increased Glut4 and transferrin receptor levels at the cell surface. Our results are in line with these data and suggest that TZDs may regulate adiponectin release via reduction of FIP1 protein. However, whether PPARγ exerts its effects on FIP1 gene directly or indirectly remains to be further investigated. At this time further experiments are needed to confirm this hypothesis.

We show that FIP1 expression was reduced in subcutaneous adipose tissue of obese subjects. In this study, FIP1 mRNA was inversely correlated with BMI, leptin and fasting triglyceride levels, reflecting a reduced expression of FIP1 in mature lipid-loaded adipocytes, in line with our *in vitro* adipogenesis data. Obesity is associated with compromised adiponectin synthesis and release [1]. However, the molecular mechanisms that lead to the regulation of FIP1 gene in human adipose tissue are currently unknown and warrant further investigation. During obesity an increased lipid accumulation and adipose cell expansion leads to adipose tissue hypoxia and subsequent adipose tissue dysfunction characterized by metabolic abnormalities, and significant alterations in the overall adipokine tissue profile. In obese adipose tissue, increased macrophage infiltration and activation leads to enhanced production and release of pro-inflammatory cytokines. We tested the hypothesis that proinflammatory cytokines could act on adipose tissue to modulate the expression of FIP1. We found that *in vitro* treatment of human or mouse adipocytes with tumor necrosis factor alpha (TNFα) or with LPS-stimulated macrophage-conditioned media, enriched with pro-inflammatory cytokines, increased FIP1 expression at the same time that it decreased adiponectin levels. Macrophage-conditioned media also increased the expression of interleukin 6 (IL-6) in human adipocytes (data not shown), however we did not see any effect of IL-6 on FIP1 expression in mouse adipocytes. Our findings agree with previous reports that showed TNFα downregulates adiponectin mRNA levels [34,45]. Interestingly, TNFα has been shown to downregulate the transcriptional activator PPARγ both by reducing its mRNA levels [34] and by reducing its transcriptional activity, causing serine phosphorylation of key regulatory residues in its N terminal domain [46,47]. While we did not directly examine the molecular mechanisms by which TNFα induced FIP1 expression it is tempting to speculate that it may be mediated in part by inhibition of PPARγ.

Hypoxia induces changes in the expression of many adipokines and has been recently shown to directly reduce the secretion of adiponectin in adipocytes [48]. In our study we found a tendency for FIP1 mRNA levels in 3T3L1 adipocytes to increase when cultured in hypoxic conditions, although the differences observed did not reach statistical significance. Hypoxia increased the expression of pro-inflammatory cytokines including IL-6 and TNFα in primary adipose and macrophages *in vitro* [49] and decreased adiponectin mRNA levels in adipocytes [49]. Hypoxia treatment increased TNFα release in stromal cells isolated from human obese visceral adipose tissue [50]. It is possible that the hypoxia treatment in our *in vitro* study did not enhance TNFα expression to sufficient levels necessary to modulate FIP1 expression in the 3T3L1 adipocyte cell line. Nevertheless, in vivo adipose tissue hypoxia may contribute to increase FIP1 expression and impair adiponectin release indirectly via upregulation of TNFα in infiltrated or adipose tissue resident macrophages. During obesity a complex interplay of local cytokines may ultimately affect FIP1 expression in adipocytes. In future studies it will be interesting to test whether anti-inflammatory cytokines can reduce FIP1 expression and enhance adiponectin release in adipocytes.

In sum, our experimental findings demonstrate that FIP1 regulates intracellular adiponectin trafficking and secretion in adipocytes. FIP1 expression is regulated during adipogenesis, obesity and thiazolidinedione treatment. We postulate that FIP1 may be involved in retaining adiponectin within the endosomal system of adipocytes or between the ERC and TGN. Further investigation is needed to elucidate the molecular basis for this action and to identify the downstream molecular partners for FIP1.

## Supporting Information

Figure S1 **Expression of FIP-GFP proteins in HEK293.** Plasmids expressing GFP-tagged wild type RAB11-FIPs and myc-tagged adiponectin were transiently co-transfected in HEK293 cells as indicated in the methods section. 24hr following transfection cell lysates were obtained and separated by SDS-PAGE, transferred to a nitrocellulose filter and immunoblotted with an anti-FIP antibody or tubulin as indicated. Lanes1-2: cells expressing FIP1-GFP; lanes 3-4: cells expressing FIP2-GFP; lanes 5-6: cells expressing FIP3-GFP; lanes 7-8; cells expressing FIP4-GFP; lanes 9-10: cells expressing FIP5-GFP. Representative blot of three independent experiments.
(TIF)

Figure S2 **Adiponectin secretion in cells expressing single or paired FIP1 shRNA** **construct**s. HEK293 cells were transfected with single FIP shRNA constructs or in paired combinations as described in the methods section. Transfected cells were selected in the presence of 5 µg/ml of puromycin for 3 days. Cells were then transfected with a plasmid coding for adiponectin-myc and 20 hrs following transfection the media and cellular lysates were harvested and the amount of adiponectin quantitated by ELISA as indicated in the methods section. Controls: cells transfected only with the plasmid coding for adiponectin-myc, cells transfected with adiponectin-myc and the shRNA empty vector or cells transfected with the pool of FIP1 shRNAs in combination ’shRNA pool’. **A**) Adiponectin secretion by ELISA. The graph shows the mean + SEM from data collected in two independent experiments, with 3-4 biological replicates, each sample quantified in duplicate by ELISA as described in the materials and methods section. Statistical Analysis: One way ANOVA, * indicates statistical significance at p<0.05. **B**) Western blot analysis of whole cell lysates of HEK293 cells transfected with single or pair combinations of FIP1 shRNA constructs or all FIP1 shRNA constructs as a pool. Lysates were obtained HEK293 expressing the shRNA empty vector (lane1), expressing single FIP1 shRNA constructs or in pair combination (lanes 2 to 10) or expressing all the shRNAs for FIP1 as a pool (lanes11 and 12). Protein samples were separated on SDS-PAGE, transferred to nitrocellulose filters and immunoblotted with specific antibodies for FIP1and actin as loading control. The data shows a representative blot of two independent experiments.
(TIF)

Figure S3 **Colocalization of endogenous adiponectin with rab14 in 3T3L1 adipocytes.** 3T3L1 control or cells expressing shRNA for FIP1 were differentiated to adipocytes as indicated in the methods section. Cells were fixed, permeabilized and stained with an antibody against rab14 or adiponectin and Alexa-488 and Alexa-594 conjugated antibodies. Representative cells are shown of two independent experiments. Determination of colocalization was carried out as described in the methods section. Control (n = 6), FIP1 shRNA (n = 4).
(TIF)

Figure S4 **Effects of hypoxia on FIP1 expression in 3T3L1 adipocytes.** 3T3L1 cells were cultured and differentiated as indicated in the methods section. Following differentiation a subset of cells was placed into an hypoxia incubator (hypoxystation) with 1% oxygen 5% CO_2_ and 94% nitrogen for either 8h, 16h or 24 hours. Total RNA was isoladed and the amount of mRNA for FIP1, adiponectin or Glut1 were determined by real time PCR. Quantification was carried out using the ∆∆Ct method using cyclophyllin A as internal control. Data from one experiment carried out in triplicate cell dishes for each condition.
(TIF)
