# Correction: The Rab11 Effector Protein FIP1 Regulates Adiponectin Trafficking and Secretion

**DOI:** 10.1371/journal.pone.0100433

**Published:** 2014-06-11

**Authors:** The PLOS ONE Staff

There is an affiliation missing for the third and fourth authors. Jose Maria Moreno-Navarrete and Jose Manuel Fernandez-Real are both affiliated with: CIBERobn Fisiopatología de la obesidad y Nutrición and IDIBGI, Girona, Spain.
