# Supplementary figures and images for: The Rab11 Effector Protein FIP1 Regulates Adiponectin Trafficking and Secretion
Source: PLoS One. 2013 Sep 11;8(9):e74687. doi: 10.1371/journal.pone.0074687 (PMC3770573; doi:10.1371/journal.pone.0074687)

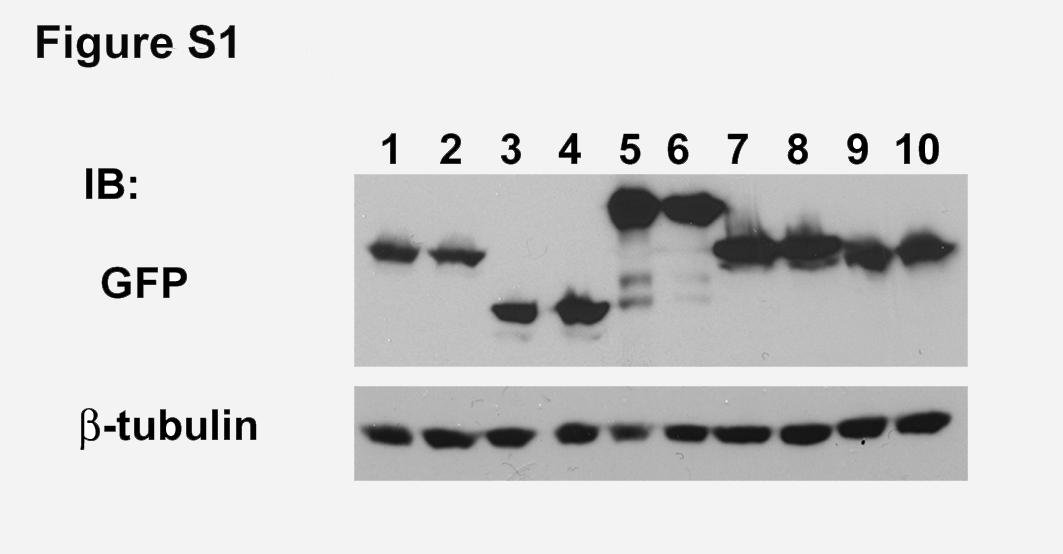

Supplement: Figure S1 — Expression of FIP-GFP proteins in HEK293. Plasmids expressing GFP-tagged wild type RAB11-FIPs and myc-tagged adiponectin were transiently co-transfected in HEK293 cells as indicated in the methods section. 24hr following transfection cell lysates were obtained and separated by SDS-PAGE, transferred to a nitrocellulose filter and immunoblotted with an anti-FIP antibody or tubulin as indicated. Lanes1-2: cells expressing FIP1-GFP; lanes 3-4: cells expressing FIP2-GFP; lanes 5-6: cells expressing FIP3-GFP; lanes 7-8; cells expressing FIP4-GFP; lanes 9-10: cells expressing FIP5-GFP. Representative blot of three independent experiments. (TIF) [file pone.0074687.s001.tif]

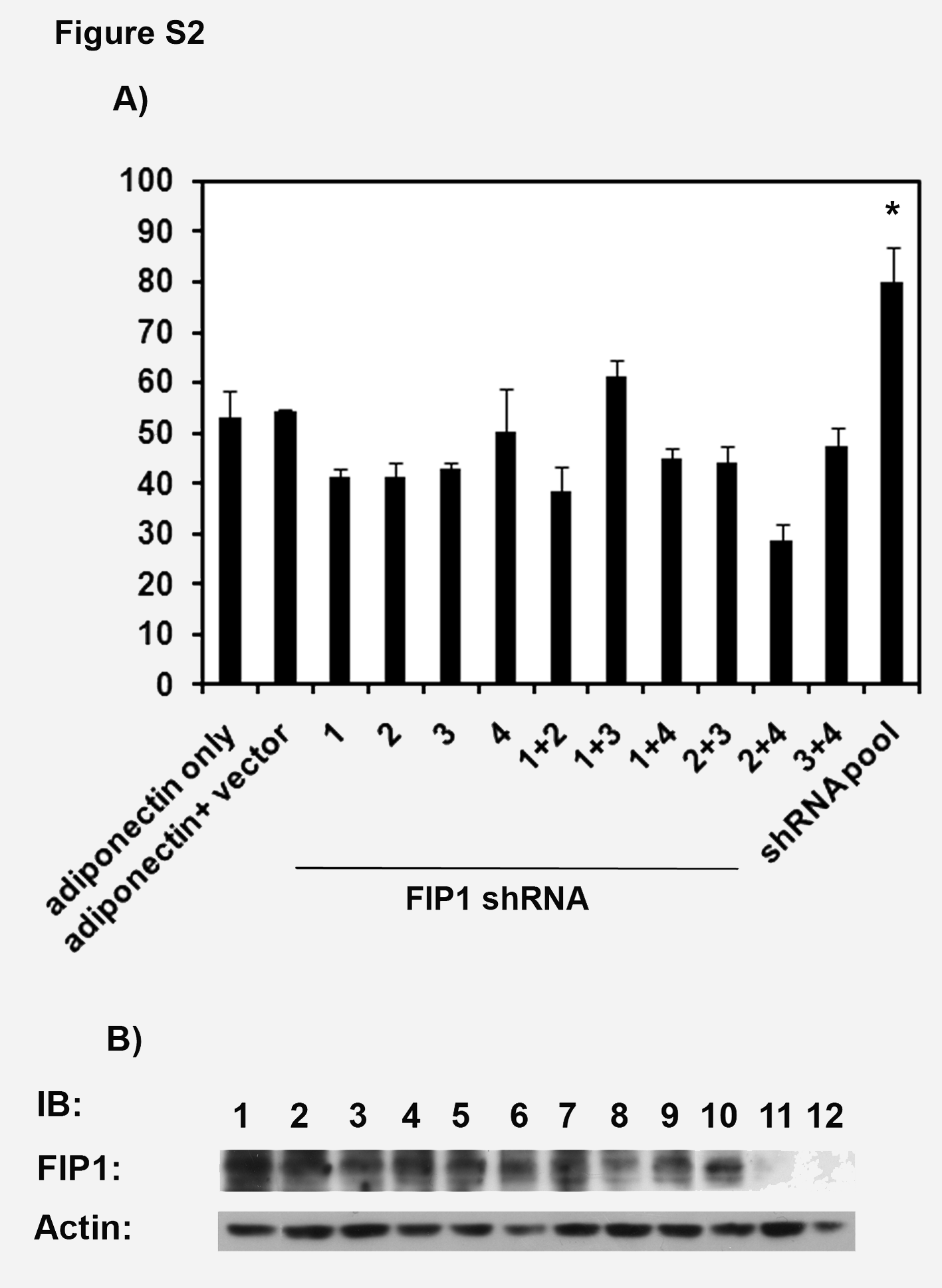

Supplement: Figure S2 — Adiponectin secretion in cells expressing single or paired FIP1 shRNA constructs. HEK293 cells were transfected with single FIP shRNA constructs or in paired combinations as described in the methods section. Transfected cells were selected in the presence of 5 µg/ml of puromycin for 3 days. Cells were then transfected with a plasmid coding for adiponectin-myc and 20 hrs following transfection the media and cellular lysates were harvested and the amount of adiponectin quantitated by ELISA as indicated in the methods section. Controls: cells transfected only with the plasmid coding for adiponectin-myc, cells transfected with adiponectin-myc and the shRNA empty vector or cells transfected with the pool of FIP1 shRNAs in combination ’shRNA pool’. A) Adiponectin secretion by ELISA. The graph shows the mean + SEM from data collected in two independent experiments, with 3-4 biological replicates, each sample quantified in duplicate by ELISA as described in the materials and methods section. Statistical Analysis: One way ANOVA, * indicates statistical significance at p<0.05. B) Western blot analysis of whole cell lysates of HEK293 cells transfected with single or pair combinations of FIP1 shRNA constructs or all FIP1 shRNA constructs as a pool. Lysates were obtained HEK293 expressing the shRNA empty vector (lane1), expressing single FIP1 shRNA constructs or in pair combination (lanes 2 to 10) or expressing all the shRNAs for FIP1 as a pool (lanes11 and 12). Protein samples were separated on SDS-PAGE, transferred to nitrocellulose filters and immunoblotted with specific antibodies for FIP1and actin as loading control. The data shows a representative blot of two independent experiments. (TIF) [file pone.0074687.s002.tif]

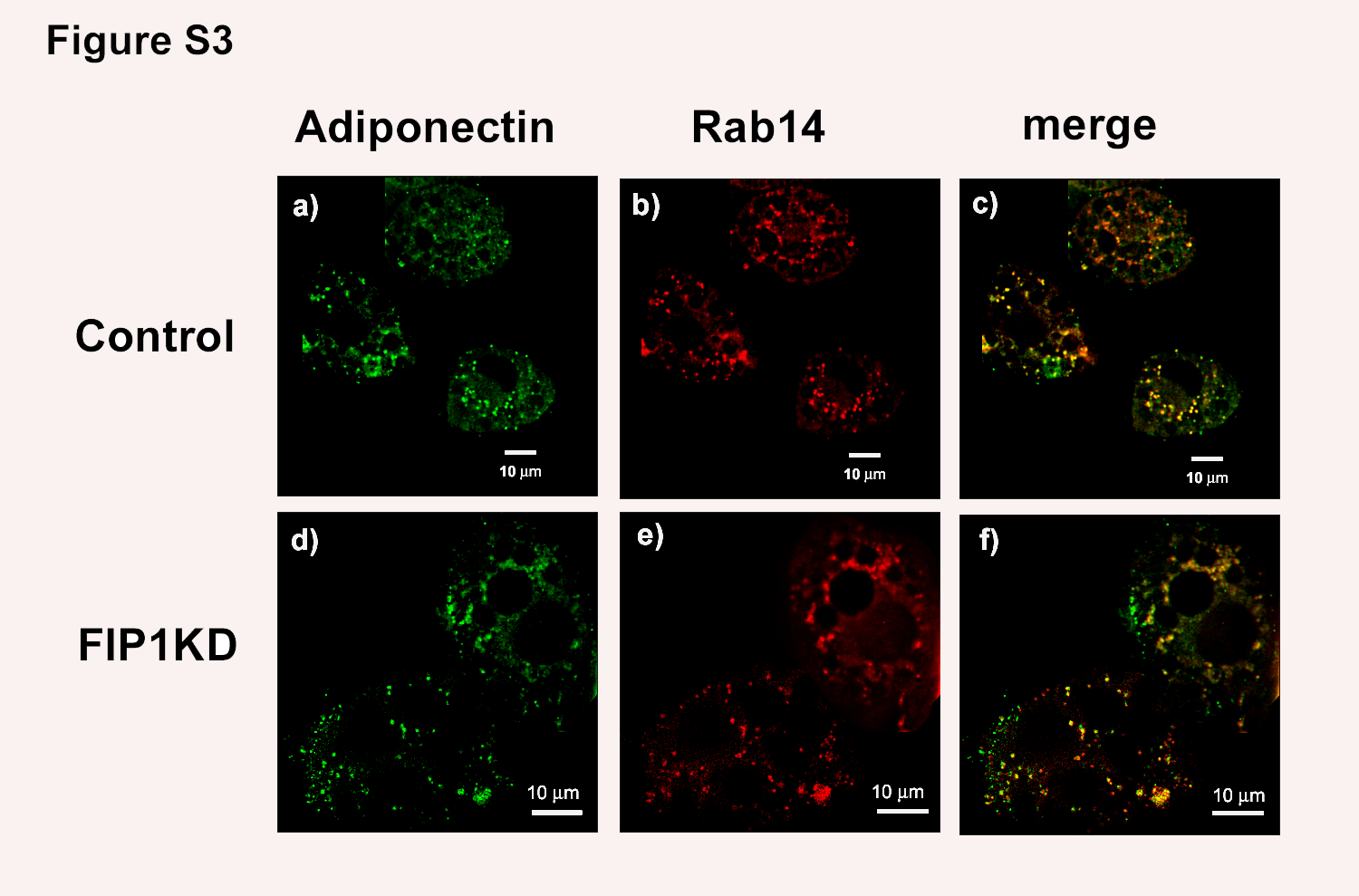

Supplement: Figure S3 — Colocalization of endogenous adiponectin with rab14 in 3T3L1 adipocytes. 3T3L1 control or cells expressing shRNA for FIP1 were differentiated to adipocytes as indicated in the methods section. Cells were fixed, permeabilized and stained with an antibody against rab14 or adiponectin and Alexa-488 and Alexa-594 conjugated antibodies. Representative cells are shown of two independent experiments. Determination of colocalization was carried out as described in the methods section. Control (n = 6), FIP1 shRNA (n = 4). (TIF) [file pone.0074687.s003.tif]

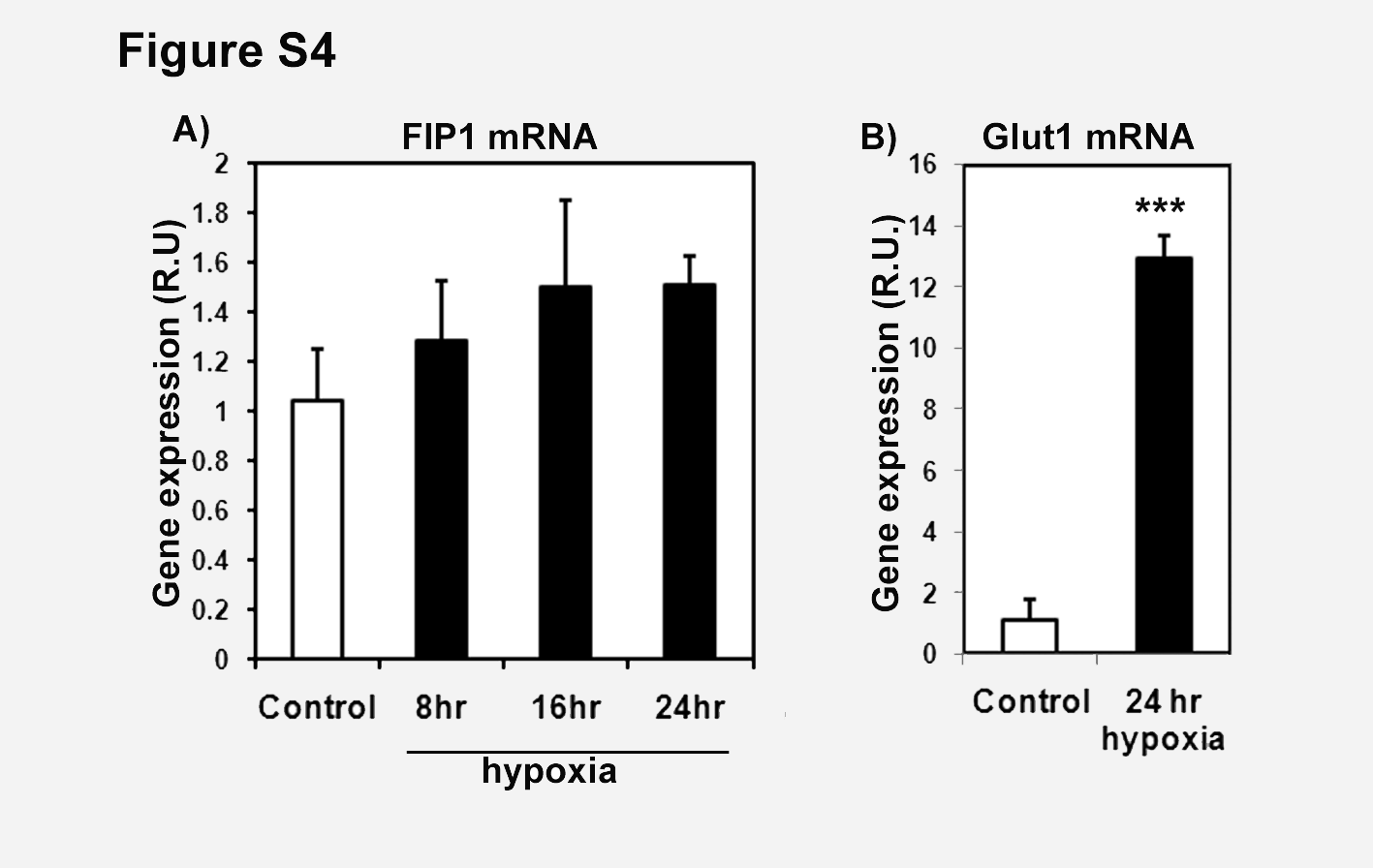

Supplement: Figure S4 — Effects of hypoxia on FIP1 expression in 3T3L1 adipocytes. 3T3L1 cells were cultured and differentiated as indicated in the methods section. Following differentiation a subset of cells was placed into an hypoxia incubator (hypoxystation) with 1% oxygen 5% CO2 and 94% nitrogen for either 8h, 16h or 24 hours. Total RNA was isoladed and the amount of mRNA for FIP1, adiponectin or Glut1 were determined by real time PCR. Quantification was carried out using the ∆∆Ct method using cyclophyllin A as internal control. Data from one experiment carried out in triplicate cell dishes for each condition. (TIF) [file pone.0074687.s004.tif]
